# Upregulation of CD14 in mesenchymal stromal cells accelerates lipopolysaccharide-induced response and enhances antibacterial properties

**DOI:** 10.1016/j.isci.2022.103759

**Published:** 2022-01-12

**Authors:** Matthew P. Hirakawa, Nikki Tjahjono, Yooli K. Light, Aleyna N. Celebi, Nisa N. Celebi, Prem Chintalapudi, Kimberly S. Butler, Steven S. Branda, Raga Krishnakumar

**Affiliations:** 1 Systems Biology Department, Sandia National Laboratories, Livermore, CA 94551, USA; 2 Molecular and Microbiology Department, Sandia National Laboratories, Albuquerque, NM 87185, USA; 3 Molecular and Microbiology Department, Sandia National Laboratories, Livermore, CA 94551, USA

**Keywords:** Molecular biology, Cell biology, Omics

## Abstract

Mesenchymal stromal cells (MSCs) have broad-ranging therapeutic properties, including the ability to inhibit bacterial growth and resolve infection. However, the genetic mechanisms regulating these antibacterial properties in MSCs are largely unknown. Here, we utilized a systems-based approach to compare MSCs from different genetic backgrounds that displayed differences in antibacterial activity. Although both MSCs satisfied traditional MSC-defining criteria, comparative transcriptomics and quantitative membrane proteomics revealed two unique molecular profiles. The antibacterial MSCs responded rapidly to bacterial lipopolysaccharide (LPS) and had elevated levels of the LPS co-receptor CD14. CRISPR-mediated overexpression of endogenous CD14 in MSCs resulted in faster LPS response and enhanced antibacterial activity. Single-cell RNA sequencing of CD14-upregulated MSCs revealed a shift in transcriptional ground state and a more uniform LPS-induced response. Our results highlight the impact of genetic background on MSC phenotypic diversity and demonstrate that overexpression of CD14 can prime these cells to be more responsive to bacterial challenge.

## Highlights

• MSCs from different genetic backgrounds have distinct responses to bacteria
• Upregulating CD14 in MSCs enhances LPS-induced response and antibacterial traits
• CD14 upregulation homogenizes MSC transcriptional profiles across individual cells

## Graphical abstract

**Figure:**
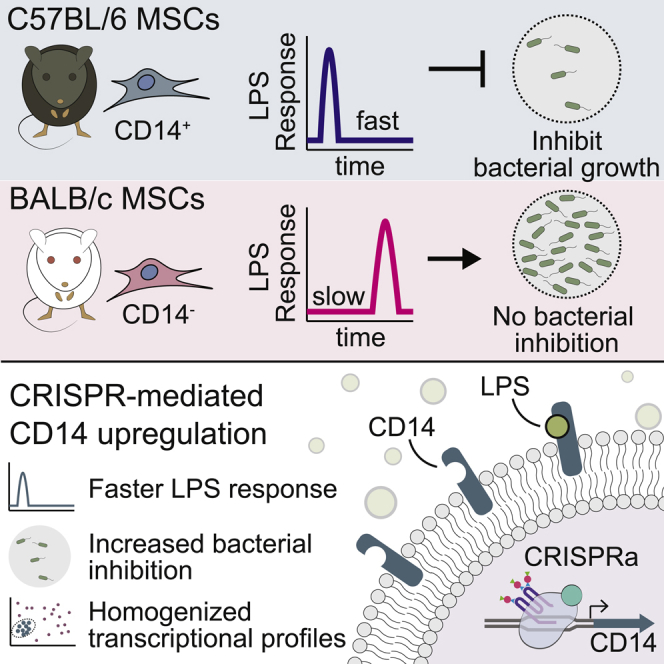

## Introduction

The emergence of drug-resistant microorganisms is a significant threat to human health that requires the development of new and alternative therapies. Mesenchymal stromal cells (MSCs) are intriguing candidates as therapeutic countermeasures to combat infection because they are capable of directly inhibiting microbial growth and modulating the host immune response (Le Blanc and Davies, 2015; Alcayaga-Miranda et al., 2017; Galipeau and Sensebe, 2018; Wang et al., 2014; Pittenger et al., 2019; Sung et al., 2016; Yang et al., 2013; Krasnodembskaya et al., 2010; Gupta et al., 2012; Kim et al., 2016a). MSCs are a group of multipotent and phenotypically plastic cells that have enormous potential for use in diverse clinical applications (Pittenger et al., 2019; Galipeau and Sensebe, 2018; Wilson et al., 2019). The antibacterial properties of MSCs are, in part, due to the secretion of antimicrobial peptides, including cathelicidin LL-37, β-defensin-2, lipocalin-2, and hepcidin, and the administration of MSCs has been shown to promote resolution of microbial infection in animal models (Krasnodembskaya et al., 2010; Sung et al., 2016; Gupta et al., 2012; Alcayaga-Miranda et al., 2015; Sutton et al., 2016; Yang et al., 2013). MSCs have the potential for broad-acting cell-based antimicrobial therapy; however, the mechanistic basis for antimicrobial activity, and the molecular tools and strategies required to generate and maintain MSC populations of uniform phenotype, a requirement for their safe and reliable use in clinical applications, are not fully understood.

One such strategy to direct, or enhance, MSC therapeutic properties is through “priming” these cells by exposing them to specific stimuli or growth conditions (Yin et al., 2019; Noronha et al., 2019; Pittenger et al., 2019; Redondo-Castro et al., 2018; Showalter et al., 2019; Qiu et al., 2017b; Park et al., 2016, 2020; Najar et al., 2017; Luo et al., 2019; Liu et al., 2017b; Bartosh et al., 2010; Ho et al., 2016). In addition, priming MSCs can often elicit population-normalizing effects that unify cellular behavior in these notoriously heterogeneous cells (Jin et al., 2016; Freeman et al., 2015; Sun et al., 2020; Wolock et al., 2019; Pittenger et al., 2019). Priming MSCs using Toll-like receptor (TLR) agonists are particularly notable, as stimulation of these receptors leads to diverse phenotypic alterations to MSCs (Pevsner-Fischer et al., 2007; Najar et al., 2017; Sangiorgi and Panepucci, 2016; Tomchuck et al., 2008; Liotta et al., 2008; Yan et al., 2014). For example, stimulation of TLR3 causes MSCs to exhibit enhanced migration, upregulates immunosuppressive traits, and provides therapeutic benefits for colitis (Waterman et al., 2010; Qiu et al., 2017b). In addition, systemic administration of TLR3-primed MSCs in animals with chronic bacterial infections can promote dramatic reduction in bacterial burden at sites of infection and promote wound healing (Johnson et al., 2017). Interestingly, although TLR3 priming tends to drive MSCs toward an immunosuppressive state, TLR4 priming shifts MSCs into a more proinflammatory state (Waterman et al., 2010; Park et al., 2016; Tomchuck et al., 2008). TLR4, together with the co-receptor CD14, recognizes bacterial lipopolysaccharide (LPS), which triggers a signal transduction cascade converging on the transcription factor NF-κB to elicit a host immune response (Park and Lee, 2013; Lu et al., 2008; Zanoni and Granucci, 2013; Liu et al., 2017a; Kawasaki and Kawai, 2014). TLR4 priming of MSCs by exposure to LPS causes widespread transcriptional changes primarily regulated by NF-κB and IRF1 and leads to the upregulation of genes involved with inflammatory response and chemotaxis (Kim et al., 2016b). Interestingly, TLR4 also serves as a critical receptor involved with antibacterial activity in MSCs, and knockdown of this protein reduces the ability of MSCs to inhibit bacterial growth (Sung et al., 2016). The role of TLR4 in regulating antibacterial properties in MSCs was further supported by experiments demonstrating that LPS-primed MSCs have enhanced antibacterial properties due to the increased expression of antimicrobial peptides (Krasnodembskaya et al., 2010; Sung et al., 2016; Saeedi et al., 2019).

Although priming studies in MSCs have demonstrated many notable therapeutic enhancements, these approaches can have pitfalls that include variability of priming effects from different MSC sources, immunogenicity complications, and extended *in vitro* expansion (Noronha et al., 2019; Yin et al., 2019). An alternative strategy to develop MSCs with enhanced therapeutic properties is to utilize targeted genetic modification strategies informed by molecular signatures identified from priming studies. Recently, there has been an increased focus on engineering MSCs to maximize therapeutic potential, and new genetic engineering tools, such as CRISPR-based genetic modifiers, may enable new approaches for optimizing these cells (Damasceno et al., 2020; Filho et al., 2019; Lee et al., 2020; Golchin et al., 2020). Such optimization also requires an adequate systems biology understanding of the developmental, transcriptomic, and proteomic states of heterogeneous MSC populations.

In this work, we examine MSCs from the bone marrow of two different mouse strains (C57BL/6 and BALB/c) for their ability to inhibit bacterial growth, with C57BL/6 MSCs (C57-MSCs) showing significantly stronger antimicrobial activity compared with BALB/c MSCs (BALB-MSCs). Using a combination of functional assays and molecular profiling strategies, we identify striking differences between these two MSC subtypes regarding their ability to sense and respond to bacterial stimulation. Based on these analyses, we focus on the LPS co-receptor CD14, which was expressed at higher levels in C57-MSCs. Using CRISPR-activation (CRISPRa) to upregulate CD14 in MSCs, we were able to enhance their ability to inhibit bacterial growth, even in the absence of a priming agent. We further characterize CD14-activated BALB-MSCs at the single-cell transcriptional level and demonstrate that CD14 expression potentiates a more rapid response to bacteria-derived LPS and reduces the transcriptional heterogeneity among the cell population. Overall, we demonstrate that a systems biology approach can reveal critical targets for enhancing specific MSC phenotypes and that CRISPR-based gene modulation is an effective strategy to engineer MSCs with potential therapeutic properties.

## Results

### MSCs from different genetic backgrounds exhibit distinct antibacterial phenotypes

The cells used in this study were commercially available bone-marrow-derived MSCs isolated from C57BL/6 or BALB/c mice and were examined for their expression of established mouse MSC surface markers and their ability to differentiate (Hu et al., 2018). We first examined the expression levels of mouse MSC markers in C57-MSCs and BALB-MSCs and observed that both cell types exhibited similar levels of these proteins using immunostaining followed by flow cytometry (Figure S1). These cells were also inspected for their ability to differentiate into adipocytes and osteoblasts, and both C57-MSCs and BALB-MSCs could differentiate under adipogenic and osteogenic inducing conditions (Figure S2) (Pittenger et al., 1999). Using these traditional metrics to define cell identity, both C57-MSCs and BALB-MSCs could be considered the same cell type.

**Figure 1:**
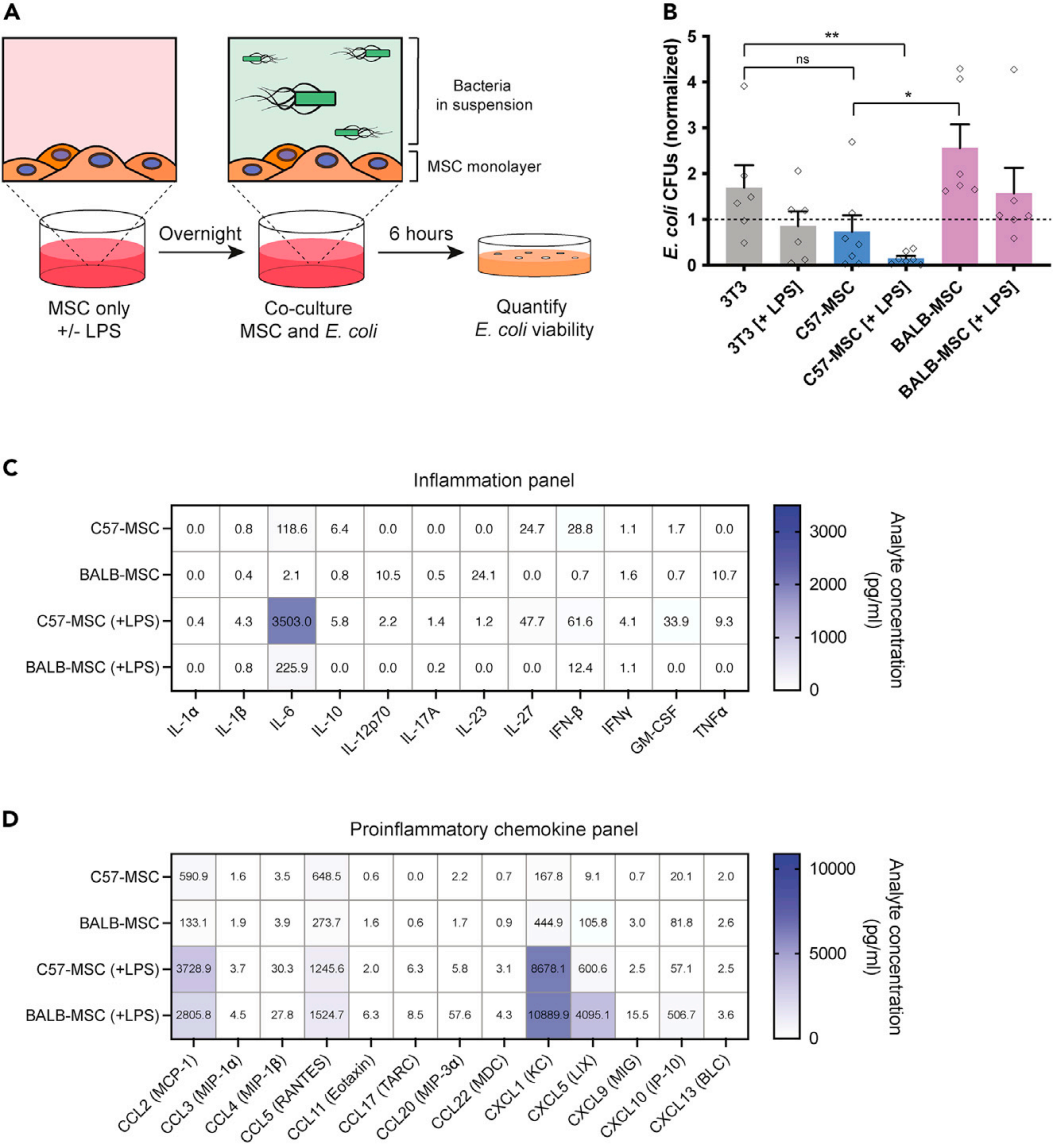
MSCs from different genetic backgrounds have distinct antibacterial properties (A) Cartoon depicting the antibacterial assays used in this study. MSCs were incubated overnight with or without lipopolysaccharide (LPS) from *E. coli* (100 ng/mL) and were then co-cultured with *E. coli* strain K12 for 6 h. After 6 h of co-culture, *E. coli* abundance in the media was quantified using colony-forming unit (CFU) assays. (B) Quantification of *E. coli* CFUs following 6-h co-culture with different cell types +/− LPS treatment. *E. coli* CFU values from co-culture experiments were normalized to controls in which *E. coli* was grown side-by-side in monoculture (n = 5 biological replicates per condition; statistical significant determined using t test; ∗p < 0.05; ∗∗p < 0.01; individual data points are shown and bar graphs represent the mean with error bars = SEM). (C and D) Cytokine profiling of C57-MSC and BALB-MSC conditioned media with or without LPS-priming using BioLegend LEGENDplex bead-based immunoassays with the mouse inflammation panel or (D) proinflammatory chemokine panel. Samples were analyzed 18-h post-LPS-exposure, and the values shown on heatmap are the mean of at least two biological replicates.

Although these C57-MSCs and BALB-MSCs are nominally the same cell type, we observed them to exhibit strikingly different phenotypes when co-cultured with bacteria. Here, we tested the ability of MSCs to limit bacterial growth by co-culturing these cells with *Escherichia coli* strain K-12 MG1655 for 6 h (referred to as *E. coli* henceforth) *in vitro* (Figures 1A and 1B). *E. coli* were also co-cultured with 3T3 cells (a mouse embryonic fibroblast [MEF]-derived cell line) because these cells have previously been utilized as a non-antibacterial mammalian cell control in a related study, and similar to previous findings, these cells were not observed to have an inhibitory effect on bacterial growth here (Gupta et al., 2012). To account for any technical variables that impacted the endpoint bacterial abundance between experiments, data were normalized to *E. coli* monoculture controls that were performed alongside every experimental replicate. Because priming MSCs via exposure to bacteria, or bacteria-derived LPS, has been shown to induce antibacterial properties in these cells, we tested whether priming 3T3s with LPS from *E. coli* 055:B5 could similarly induce an antibacterial response (Krasnodembskaya et al., 2010; Sung et al., 2016; Saeedi et al., 2019). When 3T3 cells were primed by LPS-exposure, we observed a 49% reduction in *E. coli* CFUs when compared with unprimed 3T3 cells; however, this difference was not statistically significant (p = 0.18, t test). When *E. coli* were co-cultured with C57-MSCs, we observed a 57% reduction in CFUs compared with *E. coli* grown with 3T3 cells, but this result also did not reach statistical significance (p = 0.13, t test). However, when C57-MSCs were primed with LPS before bacterial co-culture, we observed a dramatic reduction in bacterial abundance with 92% fewer *E. coli* CFUs recovered when compared with the 3T3 cell control, and an 83% reduction when compared with 3T3 cells primed with LPS (p = 0.006 and p = 0.037, respectively; t test). To test if these antibacterial properties required direct contact between *E. coli* and the C57-MSCs, antibacterial assays were performed using transwell inserts to physically separate the two cell types (Figure S3). Here, we observed that C57-MSCs were also capable of inhibiting *E. coli* growth even when bacteria were physically separated from MSCs by a transwell membrane. Alternatively, BALB-MSCs did not elicit any antibacterial activity, and *E. coli* abundance when cultured with BALB-MSCs with and without LPS-priming was not significantly different compared with 3T3 controls (p = 0.25 and p = 0.22, respectively). Interestingly, when *E. coli* were co-cultured with BALB-MSCs we observed a 251% increase in bacterial abundance compared with *E. coli* co-cultured with C57-MSCs (p = 0.012, t test). Together, these data demonstrate that although MSCs isolated from different genetic backgrounds share hallmark features of MSCs, their antibacterial properties are distinct, which may have important implications regarding the efficacy of MSCs as potential therapy to treat bacterial infections.

To determine if LPS priming led to differential cytokine production between C57-MSCs and BALB-MSCs, conditioned media was collected from cells in the absence of LPS or after 18 hours of LPS exposure. Cytokine profiles were examined using the inflammation panel and the proinflammatory chemokine panel of the BioLegend LEGENDplex bead-based immunoassay platform. Both cell types exhibited similar inflammatory cytokine profiles with many of the cytokines assayed being low or below the limit of detection in the absence of LPS (Figure 1C). The most significant difference observed between cell types was the production of interleukin-6 (IL-6), with levels being ∼60-fold higher in C57-MSCs (p = 0.0079, t test). IL-6 levels remained elevated in C57-MSCs after LPS-exposure and exhibited ∼16-fold more IL-6 compared with BALB-MSCs. The abundance of proinflammatory chemokines were also measured, and similar to the inflammation panel, most of the chemokines in the panel were not detected or were observed at low levels when no LPS was added (Figure 1D). Without LPS stimulation, there was a ∼4-fold higher level of CCL2 (MCP-1) in C57-MSC conditioned media compared with BALB-MSC media (p = 0.0042, t test). After LPS treatment, both MSC types exhibited elevated levels of CCL2, CCL5, and CXCL1 (>1 ng/mL), and we observed ∼7-fold higher levels of CXCL5 produced by BALB-MSCs compared with C57-MSCs. Together, these results prompted us to further investigate the molecular mechanisms contributing to the differences in C57-MSC versus BALB-MSC LPS response and antibacterial activity.

### C57-MSCs exhibit more rapid transcriptional response to LPS stimulation than BALB-MSCs

**Figure 2:**
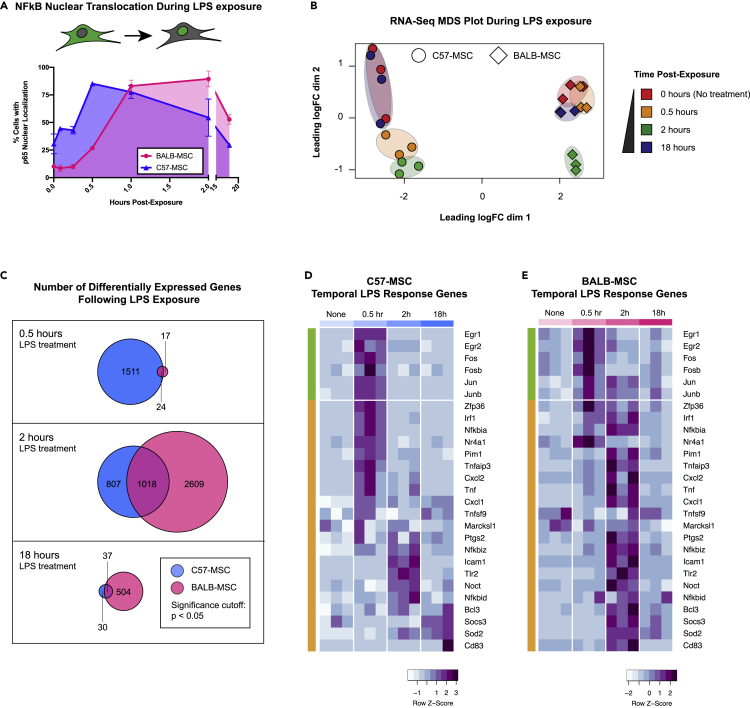
Differential response rates to LPS between MSCs from different genetic backgrounds (A) Quantification of NF-κB nuclear translocation assays using p65 staining and high-throughput imaging (>1000 cells analyzed per replicate; n = 3 biological replicates; error bars represent SD). (B) MDS plot depicting transcriptional profiles of C57-MSCs and BALB-MSCs at time points post-LPS-exposure (0, 0.5, 2, and 18 h). (C) Numbers of differently expressed genes at different time points post-LPS-exposure compared with corresponding untreated cells. (D and E) Heatmap depicting temporal expression of immediate early genes (green bars) and primary LPS response (orange bars) in C57-MSC and BALB-MSC at time points post-LPS-exposure (immediate-early genes and primary LPS response genes were previously defined (Bahrami and Drablos, 2016; Fowler et al., 2011; Ramirez-Carrozzi et al., 2009). For each time point analyzed, three biological replicates were sequenced and are depicted by sub-columns in the heatmap.

LPS exposure triggers a number of signaling pathways in mammalian cells, leading to the nuclear translocation of cytoplasmic sequestered transcription factors, including the NF-κB family of transcription factors that are key regulators of antimicrobial activity and inflammation (Lu et al., 2008; Palsson-McDermott and O'Neill, 2004; Napetschnig and Wu, 2013; Liu et al., 2017a). We hypothesized that the absence of antibacterial activity exhibited by BALB-MSCs may be the result of an attenuated or delayed recognition of bacterial exposure when compared with the C57-MSC response. To examine temporal responses to LPS stimulation, C57-MSCs and BALB-MSCs were treated with LPS, and nuclear translocation of the NF-κB subunit p65 was assayed using immunostaining followed by quantitative high-throughput microscopy (Figures 2A and S4). In these assays, C57-MSCs responded to LPS stimulation with a peak nuclear p65 localization (∼85% of cells analyzed) occurring at 0.5 h postexposure. In contrast with the C57-MSCs, only ∼27% of BALB-MSCs displayed nuclear p65 at the 0.5 h time point (p = 3 × 10^−6^). Instead, the BALB-MSCs exhibited peak nuclear p65 staining (∼90% of cells) occurring at 2 h postexposure, indicating that BALB-MSCs were somehow slower to transduce the LPS signal from the cell surface into the nucleus when compared with C57-MSCs.

Because we observed C57-MSCs and BALB-MSCs to exhibit distinct NF-κB nuclear translocation kinetics in response to LPS, we hypothesized that rates of transcriptional change would also differ in these two cell types. To examine patterns of gene expression following LPS exposure, population-level transcriptional profiling (RNA-seq) was performed on MSCs before LPS exposure and at three time points following LPS exposure (0.5, 2, and 18 h). To examine if MSCs demonstrated a transcriptional response associated with LPS stimulation, we examined the expression levels of the gene encoding the p65 subunit of NF-κB (*Rela*) as an internal control and observed significant upregulation in both C57-MSCs and BALB-MSCs after LPS exposure (Figure S5). To visualize the relationships between MSC transcriptional profiles after LPS exposure, read-count matrices were plotted using multidimensional scaling (Figure 2B). This approach clearly separated the C57-MSCs from the BALB-MSCs along dimension 1, suggesting these two cell types have distinct transcriptional profiles. Changes in transcriptional profiles that corresponded to the time following LPS exposure were apparent along dimension 2. C57-MSCs collected at 0.5 h or 2 h postexposure formed two distinct clusters, indicating that their transcriptomes at these time points differed from that of resting-state C57-MSCs. In contrast, C57-MSCs at 18 h postexposure clustered very closely with their untreated counterparts, indicating that their transcriptome had returned to the resting state. Consistent with their slower response to LPS exposure as evident from NF-κB translocation kinetics, the BALB-MSC transcriptome at 0.5 h postexposure closely resembled that of untreated BALB-MSCs. By 2 h postexposure, the BALB-MSC transcriptome was clearly distinct from that of untreated BALB-MSCs, clustering in a similar position as C57-MSCs along dimension 2, and by 18 h postexposure returned to the resting state (i.e., resembled the untreated BALB-MSC transcriptome). These results indicate that after exposure to LPS, the C57-MSC transcriptome is rapidly altered, whereas the BALB-MSC transcriptome shows delayed response.

We next performed differential gene expression analysis to identify genes that had significant changes in expression after LPS exposure (Figure 2C). We found that 1,535 genes were differentially expressed in C57-MSCs by 0.5 h postexposure, with the majority (926 genes) being upregulated. Enriched functional gene ontology (GO) categories of these upregulated genes included regulation of adaptive immune response and cortical cytoskeleton organization. Of particular note, the genes displaying greatest fold change included those encoding chemokines *Cxcl1* and *Cxcl2*, the NF-κB inhibitor *Nfkbia*, and an RNA-binding protein involved with early LPS response (*Zfp36*). These results indicate that by 0.5 h postexposure, the C57-MCSs had mounted a multifaceted response including gene programs relevant for combating infection. In contrast, only 41 genes were differentially expressed in BALB-MSCs by 0.5 h postexposure. The small number of differentially expressed genes was not sufficient to support GO category enrichment analysis; however, several of the genes are implicated in early LPS response and were similarly differentially expressed in C57-MCs by 0.5 h postexposure (*Jun*, *Cxcl1*, *Nfkbia*, *Tnfaip*, *Zfp36*, and *Ier3*).

We further examined a subset of previously characterized early LPS response genes to better understand the timing of activation of these genes in our MSCs. These early response genes include immediate-early genes (IEGs) that are conserved across cell types and are rapidly activated by diverse environmental stimuli (Bahrami and Drablos, 2016; Fowler et al., 2011). Following IEG activation, primary LPS response genes are activated, which are more specific to this stimulus (Bahrami and Drablos, 2016; Ramirez-Carrozzi et al., 2009). Consistent with the rapid activation of IEGs following LPS stimulation, both C57-MSCs and BALB-MSCs displayed robust upregulation of the conserved IEGs *Egr1*, *Egr2*, *Fos*, *Fosb*, *Jun*, and *Junb* by 0.5 h postexposure (Figures 2D and 2E). However, the expression of primary LPS response genes varied with MSC source, with the majority showing peak expression at 0.5 h postexposure in C57-MSCs versus 2 h postexposure in BALB-MSCs. Together, these results indicate that by 0.5 h postexposure, the C57-MSCs had already progressed into an LPS-specific response, whereas the BALB-MSCs were only beginning to undergo changes in gene expression involved with generic stimulus exposure.

Finally, we examined upregulated genes at 18 h postexposure, as this is the time point at which MSCs were challenged with *E. coli* in our antibacterial assays. At this time point, both C57-MSCs and BALB-MSCs were more similar to untreated cells as compared with their transcriptional profiles at 2 h postexposure. However, there were notable genes with elevated expression that have previously been implicated in innate immune response or antibacterial activity. In particular, C57-MSCs showed increased expression of the genes encoding chemokines *Cxcl1* and *Cxcl5*, nitric oxide synthase *Nos2*, a serine protease inhibitor *Serpina3*, and the proinflammatory cytokine *Il-6*. BALB-MSCs exhibited more differentially expressed genes (541), with those upregulated (376) tending to belong to GO categories for LPS-mediated signaling and regulation of inflammatory response. Similar to C57-MSCs, among the most upregulated genes at 18 h postexposure were those involved with innate immune responses or direct antibacterial activity; these included *Nos2, Il-6*, *Cxcl1*, *Cxcl5*, the iron-sequestering protein lipocalin-2 (*Lcn2*), and complement component 3 (*C3*). Thus, by 18 h post-LPS exposure, the overall transcriptomes of C57-MSCs and BALB-MSCs very closely resembled their unstimulated counterparts; however, both cell types exhibited elevated expression of a handful of genes that are involved with recruitment of immune cells and antibacterial activity.

### C57-MSC versus BALB-MSC expression of genes mediating LPS recognition

Because C57-MSCs responded more quickly to LPS and were capable of mounting a more robust antibacterial response than BALB-MSCs, we hypothesized that the baseline transcriptional state of C57-MSCs may be different from that of BALB-MSCs to allow them to respond more quickly to bacterial exposure. To test this hypothesis, we used RNA-seq to examine the transcriptomes of unstimulated C57-MSCs and BALB-MSCs. In addition, we performed RNA-seq on two fibroblast cell types for comparison: primary mouse embryonic fibroblasts (MEFs) and primary mouse dermal fibroblasts (MDFs) from C57BL/6 mice (Figure S6). To define the relationships between cell types, transcriptional profiles were first visualized using principal component analysis (PCA). Using this approach, the C57-MSCs and BALB-MSCs clustered separately along PC1, but both diverged similarly from the fibroblasts along PC2. The C57-MSCs and BALB-MSCs shared 523 upregulated genes when compared with both fibroblasts. These genes were associated with multiple GO-term categories ascribed to innate immune response and could be summarized by a single statistically significant GO-slim category “defense response.” Of particular note, MSCs were enriched for the TLR-signaling pathway (GO:0002224) that included higher expression of genes involved with responding to bacterial PAMPs including *Lbp*, *Tlr2*, and *Irf1*. In addition, MSCs were enriched for genes involved with cell killing (GO:0031341), which included chemokines (*Cxcl1* and *Cxcl5*). Although we do not further characterize the function of these genes in this work, the genes found to be upregulated in MSCs compared with fibroblasts could be useful as additional markers for identification or isolation of MSCs.

**Figure 3:**
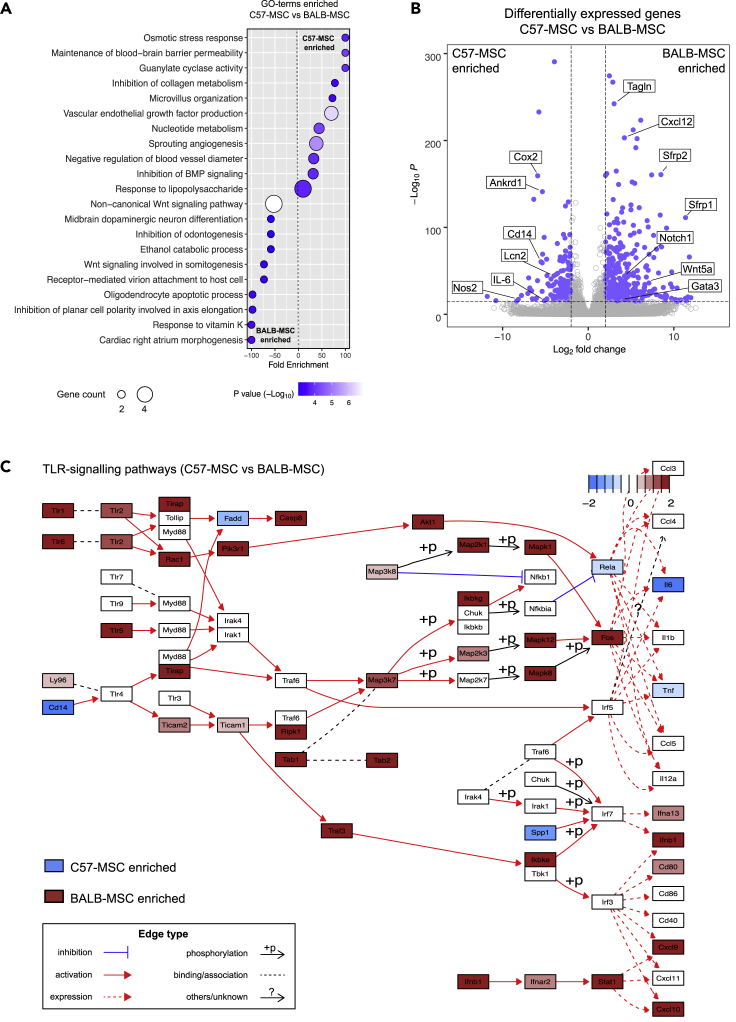
Comparative transcriptional analyses of MSC types using RNA-seq (A) RNA-seq data from C57-MSCs and BALB-MSCs were examined for differential gene expression using DeSeq2 (Love et al., 2014). Genes that were upregulated in C57-MSCs compared with BALB-MSCs (fold change >2, adjust p < 0.05) were inputted into the PANTHER GO Enrichment Analysis to find biological processes that were enriched in C57-MSCs. The same process was also performed to identify GO-terms associated with genes upregulated in BALB-MSCs. (B) Volcano plot depicting differentially expressed genes between C57-MSCs and BALB-MSCs and highlighting notable genes of interest (blue dots represent genes with fold change >2 and adjusted p-value < 10^−16^). (C) Pathway analysis visualization of comparative gene expression between C57-MSCs (blue) and BALB-MSCs (red) of TLR-signaling pathways.

To further highlight the differences between C57-MSCs and BALB-MSCs at the transcriptional level, RNA-seq data from the two MSC types were directly compared through differential expression analysis. We identified 662 genes that were upregulated in C57-MSCs relative to BALB-MSCs; and 1324 genes upregulated in BALB-MSCs relative to C57-MSCs. These genes that are specifically enriched in C57- or BALB-MSCs were then used in GO-term analysis to identify overrepresented biological functions (Figure 3A). Among the enriched GO-categories in C57-MSCs was the response to LPS, which supported our functional observations that these cells respond more quickly to LPS exposure.

We next identified the genes exhibiting the highest levels of differential expression between baseline C57-MSCs and BALB-MSCs to determine whether they could provide additional insight into differences in antibacterial activity. Interestingly, among the most upregulated genes in C57-MSCs included those involved with inflammation and antibacterial responses, including *Ptgs2* (Cox2), *Lcn2*, *Nos2*, *Il-6*, and *Cd14* (Figure 3B). These genes are all known to be regulated by NF-κB or direct activators of the NF-κB pathway, which in turn is closely tied to TLR-mediated pathogen recognition (Yamamoto et al., 1995; Ackerman et al., 2008, Kaltschmidt et al., 2002; Guo and Bhat, 2006; Morris et al., 2003; Fujino et al., 2006; Libermann and Baltimore, 1990; Zanoni and Granucci, 2013). Higher levels of secreted IL-6 protein were also observed in C57-MSCs before and after LPS treatment through cytokine profiling experiments, providing some support linking RNA-seq data with protein expression levels (Figure 1C). Among the most upregulated genes in BALB-MSCs were those involved with development and cell polarization (e.g., *Gata3*, *Notch1*, *Wnt5a*, *Sfrp1*, and *Sfrp2*), which corresponded with many of the GO-term categories enriched in these cells. Because C57-MSCs was more responsive to LPS, we hypothesized that these C57-MSCs would display higher expression levels of genes participating in TLR signaling pathways as compared with BALB-MSCs. However, using the pathway analysis tool Pathview (Luo and Brouwer, 2013), we unexpectedly observed that the majority of TLR pathway components were more highly expressed in BALB-MSCs (Figure 3C). However, a striking exception in these pathways was the elevated expression level of the LPS co-receptor *Cd14* in C57-MSCs. Because we had observed C57-MSCs to respond more rapidly to LPS stimulation and expressed higher levels of *Cd14*, we examined this gene further in subsequent sections of this work.

### Comparative membrane proteomics between cell types

**Figure 4:**
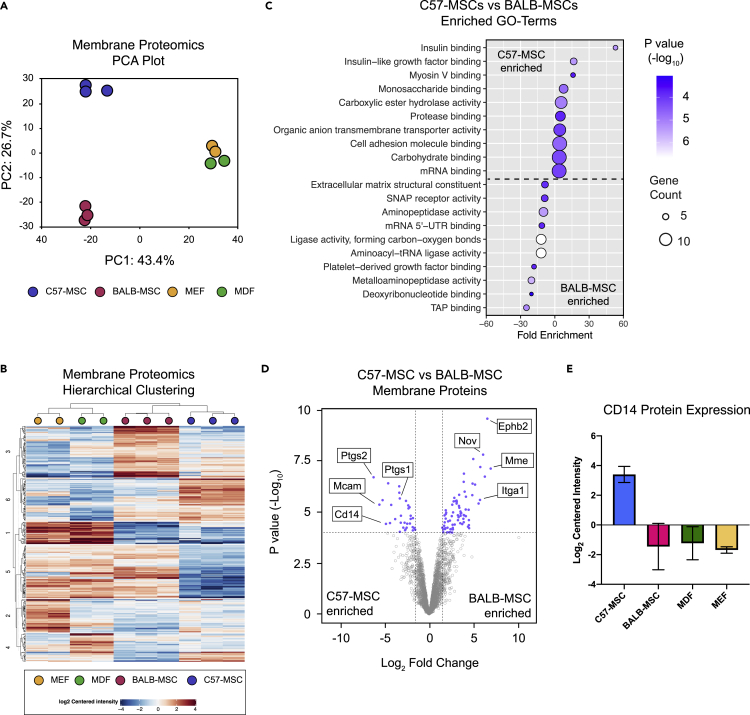
Characterization of cell membrane protein differences between cell types using quantitative proteomics MSCs and fibroblasts were grown in standard culture conditions, and membrane proteins were analyzed using LC-MS/MS (A) PCA plot depicting membrane protein profiles of MSC and fibroblast cell types (n = 3 biological replicates for MSCs and n = 2 biological replicates for MEF and MDF). (B) Heatmap utilizing hierarchical clustering showing expression level differences of membrane proteins. (C) GO-term enrichment analysis of differentially expressed membrane proteins between C57-MSCs and BALB-MSCs. MSC membrane proteins were quantified using LC-MS/MS and analyzed for differential protein expression using the DEP R package (Zhang et al., 2018). (D) Volcano plot highlighting significantly differentially expressed proteins between C57-MSCs and BALB-MSCs and notable proteins of interest (blue dots represent proteins with fold change >2 and adjusted p-value < 10^−4^). (E) Protein expression levels of the LPS-receptor CD14 across the cell types examined in this study (n = 3 biological replicates for C57- and BALB-MSCs and two biological replicates for MDFs and MEFs; data are represented as mean +/− SD).

To further investigate molecular differences between C57-MSCs and BALB-MSCs, we utilized a proteomics-based approach to characterize protein levels between these cell types using liquid chromatography-mass spectrometry (LC-MS). We focused on membrane proteins, as these would likely mediate direct interaction with bacteria and initiate downstream signaling. To visualize membrane protein profiles, label-free quantification (LFQ) intensities were plotted using PCA and hierarchical clustering to identify sample relatedness (Figures 4A and 4B). MEFs and MDFs clustered closely together, indicating that these cell types have similar membrane protein compositions compared with the MSCs. The C57-MSCs and BALB-MSCs clustered together along PC1, away from the MEFs and MDFs, but diverged from each other along PC2. Using differential protein expression analysis, we identified 1,003 proteins that showed significant differences in abundance between the MSC types, with 438 more abundant in C57-MSCs and 565 more abundant in BALB-MSCs. We next utilized GO analysis to determine if these differentially expressed proteins could point to any specific biological functions that may provide insight into their differences in antibacterial activity (Figure 4C). Among the most enriched GO categories in C57-MSCs included proteins involved with insulin binding and cell adhesion binding, whereas BALB-MSCs were enriched for TAP binding and aminopeptidase activity. We also examined the most differentially expressed membrane proteins between the two MSC types to inspect for proteins that may be associated with antibacterial phenotypic differences (Figure 4D). Of note, among the most enriched proteins in C57-MSCs was the LPS receptor CD14, consistent with RNA-seq data presented in Figure 3. CD14 protein expression was also observed in C57-MSCs, but not BALB-MSCs, using immunostaining followed by flow cytometry (Figure S7). Because CD14 is often used as a marker of human monocytes and macrophages, we queried our membrane proteomic dataset for the expression of other myeloid markers to test whether these MSCs were contaminated with myeloid lineage cells. Using this approach, we did not observe expression of any additional myeloid lineage markers in these cells. Among the most enriched proteins in BALB-MSCs were a membrane metalloendopeptidase (MME, also known as neprilysin) and ephrin type-B receptor, Ephb2. Comparing CD14 levels across all four cell types analyzed revealed that only C57-MSCs expressed high levels of CD14 protein (Figure 4E), suggesting that this protein may be critical for mounting an efficient LPS response and promoting antibacterial properties. Together, through our functional LPS response assays, comparative transcriptomic and proteomic analyses, we hypothesized that BALB-MSCs may have attenuated antibacterial properties due to a lack of CD14. In the remaining sections, we tested this hypothesis by upregulating endogenous CD14 using CRISPR tools and examined the LPS response and antibacterial properties of these engineered BALB-MSCs.

### Overexpression of endogenous CD14 enhances LPS response rate and antibacterial properties in MSCs

CD14 facilitates LPS recognition by TLR4, significantly enhancing the response to LPS in mammalian cells and in mice (Haziot et al., 1996; Perera et al., 1997). In this study, we observed BALB-MSCs and C57-MSCs to exhibit distinct LPS response kinetics, and through transcriptional and proteomic profiling we found *Cd14* was differentially regulated between these two cell types. We therefore hypothesized that the upregulation of *Cd14* could augment the attenuated antibacterial activity in BALB-MSCs. To test this hypothesis, BALB-MSCs were engineered to overexpress endogenous *Cd14*, through the use of CRISPR-mediated activation (CRISPRa). Although differential expression of *Tlr4* was not observed between cell types, overexpression of this gene was also examined independently here, as it functions in conjunction with *Cd14* and has a well-established role in propagation of LPS signaling (Palsson-McDermott and O'Neill, 2004; Kawasaki and Kawai, 2014). First, BALB-MSC-CRISPRa were constructed using lentiviral vectors to stably express the CRISPRa synergistic activation mediator (SAM) system (Konermann et al., 2015). Second, BALB-MSC-CRISPRa were tested for overexpression of target genes using six sgRNAs that localized to different distances upstream of the *Cd14* or *Tlr4* transcription start site (TSS). MSCs expressing different sgRNAs were tested for protein expression levels by immunostaining followed by flow cytometry (Figure S8). The sgRNAs that led to the most robust protein expression were used for subsequent experiments.

**Figure 5:**
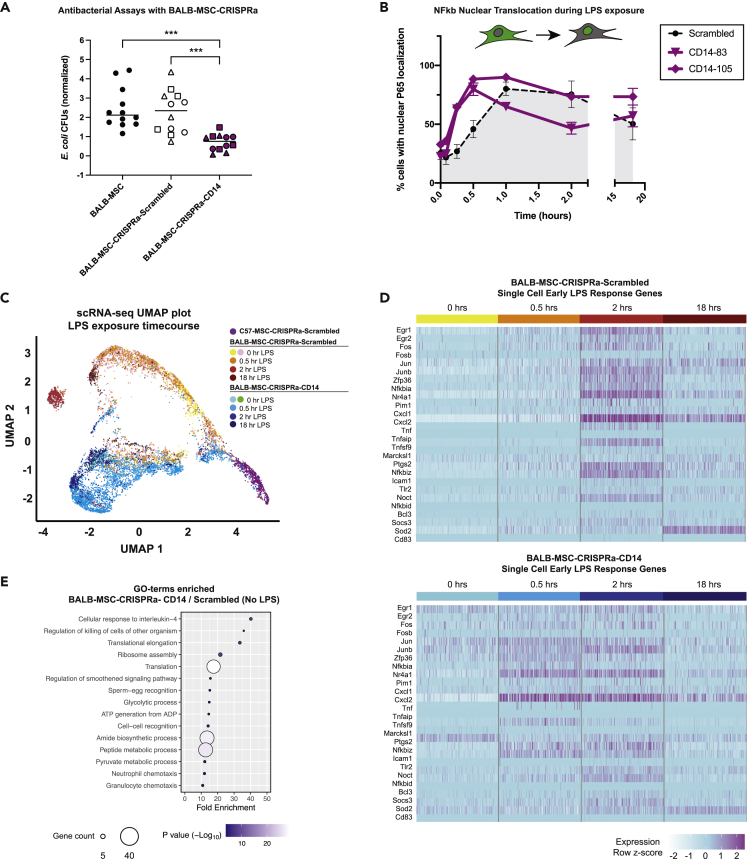
Antibacterial and single-cell transcriptional analyses of BALB-MSCs overexpressing endogenous CD14 via CRISPRa (A) *E. coli* CFUs after 6 h co-cultured with wild-type or BALB-MSC-CRISPRa cells. *E. coli* CFUs from co-cultures were normalized to *E. coli* monoculture CFU controls performed alongside each replicate. Three different sgRNAs were tested in CRISPRa MSCs, and the data from distinct sgRNAs are depicted by different shapes (for scrambled sgRNAs: circle = nontargeting sgRNA 1, square = nontargeting sgRNA 2, triangle = nontargeting sgRNA 3. For CD14 sgRNAs: circle = CD14-83, square = CD14-105, triangle = CD14-13). Lines are drawn at mean, and statistical significance was determined by t test; ∗∗∗, p < 0.001. (B) Nuclear translocation of NF-κB during LPS-exposure using p65 immunostaining and quantitative microscopy in BALB-MSC-CRISPRa cells expressing a scrambled sgRNA or CD14 sgRNAs (scrambled sgRNA = nontargeting control one; n = at least three biological replicates per sample). (C) UMAP plot depicting single-cell transcriptional profiles of MSCs during LPS-exposure (scrambled sgRNA = nontargeting control one; CD14 sgRNA = CD14-83). Single-cell RNA-seq of BALB-MSC overexpressing CD14 during LPS-exposure was prepared using the 10x Genomics Chromium platform followed by Illumina sequencing. (D) Heatmap of early LPS response gene expression in 100 individual BALB-MSC-CRISPRa cells expressing either scrambled control sgRNA (top) or CD14 sgRNA (bottom). Each sub-column represents gene expression level in an individual cell. (E) GO-terms enriched from significantly upregulated genes identified BALB-MSC-CRISPRa-CD14 when compared with BALB-MSC-CRISPRa-Scrambled in the absence of LPS-exposure.

To test how upregulation of *Cd14* or *Tlr4* impacted the antibacterial properties in BALB-MSCs, cells overexpressing these genes were co-cultured with *E. coli* and bacterial growth was quantified using CFU assays (Figure 5A). There was not a significant difference in the final *E. coli* abundance when bacteria were co-cultured with wild-type BALB-MSCs or BALB-MSCs expressing “scrambled” nontargeting control sgRNAs (p = 0.70). In contrast, when *E. coli* was grown with BALB-MSCs overexpressing *Cd14* (BALB-MSC-CRISPRa-CD14), we observed a 64% reduction in bacterial abundance compared with *E. coli* grown with wild-type BALB-MSCS and a 68% reduction compared with bacteria grown with BALB-MSC-CRISPRa expressing scrambled sgRNA controls (p = 2.7 × 10^−5^ and 2 × 10^−4^, respectively, t test). Overexpression of *Tlr4* in BALB-MSCs similarly reduced bacterial growth by 40% when compared with wild-type BALB-MSCs and 46% when compared with BALB-MSC-CRISPRa expressing scrambled nontargeting controls (p = 7 × 10^−4^ and 0.003, respectively) (Figure S9A). In addition, we constructed C57-MSCs overexpressing *Cd14* or *Tlr4* using CRISPRa and tested these cells for changes in antibacterial properties (Figure S10A). Similar to BALB-MSCs with upregulated *Cd14*, we observed that C57-MSC-CRISPRa-CD14 cells had enhanced antibacterial activity when compared with cells expressing nontargeting sgRNAs and reduced the final bacterial abundance by ∼41% (p = 0.019). However, unlike the BALB-MSC background, the upregulation of *Tlr4* in the C57-MSC background did not have a significant effect on antibacterial activity. These results demonstrate that the upregulation of endogenous *Cd14* can significantly improve antibacterial properties in MSCs, even in the absence of LPS priming.

As previously discussed, LPS stimulation triggers the translocation of NF-κB from the cytoplasm into the nucleus. To test how overexpression of *Cd14* or *Tlr4* affects the rate of NF-κB nuclear translocation, engineered BALB-MSCs were treated with LPS, and nuclear translocation of the p65 NF-κB subunit was quantified at various time points using immunostaining and quantitative high-throughput microscopy (Figure 5B). In this experiment, the BALB-MSC-CRISPRa-Scrambled control exhibited peak levels of nuclear NF-κB at 1 h post-LPS exposure. Importantly, the overexpression of *Cd14* in these BALB-MSCs caused NF-κB translocation to occur more quickly, with cells exhibiting peak levels of nuclear NF-κB at 30 min postexposure (p < 0.01). Interestingly, *Tlr4* overexpression did not significantly affect NF-κB translocation kinetics, as these cells were not different from cells expressing a nontargeting control sgRNA (Figure S9B). Similarly, we observed a faster rate of NF-κB nuclear translocation in C57-MSCs with upregulated *Cd14*, but no change in NF-κB translocation kinetics in cells with upregulated *Tlr4* when compared with control cells, suggesting that *Tlr4* is not a rate-limiting factor in the kinetics of signal transduction in this case (Figures S10B andS10C); this suggests that one mechanism by which *Cd14*-overexpressing MSCs may increase their antibacterial activity is through a faster response to bacterial stimulation.

### Overexpression of *Cd14* in BALB-MSCs increases the rate of LPS response as measured by single-cell RNA-seq

In order to more closely examine the molecular properties and kinetics of LPS response in *Cd14*-overexpressing BALB-MSCs, we performed single-cell RNA-seq using the 10X Genomics Chromium single-cell sequencing platform. BALB-MSC-CRISPRa-Scrambled and BALB-MSC-CRISPRa-CD14 were analyzed at 0, 0.5, 2, and 18 h post-LPS exposure. In addition, we examined C57-MSC-CRISPRa expressing a scrambled sgRNA in the absence of LPS as a control cell type for comparison. Using this approach, we observed that these BALB-MSC-CRISPRa-CD14 displayed accelerated LPS response kinetics when compared with those cells expressing a scrambled control. These results were observed using dimensional reduction clustering approaches, UMAP and tSNE, to visualize global transcriptional profiles of individual cells (Figure 5C, Figures S11A and S11B) (Becht et al., 2018; van der Maaten, 2014). Populations of the BALB-MSC-CRISPRa-Scrambled cells separated from the untreated cells 2 h post-LPS-exposure. These BALB-MSC-CRISPRa-Scrambled cells did not exhibit a shift in transcriptional profiles at 0.5 h post-LPS-exposure, as these two samples were highly overlapping using UMAP and tSNE clustering approaches. Alternatively, a clear separation of BALB-MSC-CRISPRa-CD14 cells was observed by 0.5 h post-LPS exposure when compared with their corresponding untreated cells, supporting the observation that these cells can respond more rapidly to LPS. This temporal LPS response was also observed using a heatmap depicting the expression levels of early LPS response genes (similar to that in Figure 2) by examining 200 individual cells per time point (Figure 5D). From these data, we observed cells expressing the scrambled guide to have highest expression levels of the majority of these genes at 2 h post-LPS-exposure. Alternatively, BALB-MSCs overexpressing *Cd14* had elevated expression of many early LPS response genes 0.5 h post-LPS exposure, consistent with the more rapid NF-κB translocation observed in these cells.

### Single-cell transcriptomic profiling of *Cd14*-overexpressing MSCs reveals a shift in ground-state and increased population homogeneity

To further examine the molecular states of the various cell types and conditions, we again examined population structure using tSNE and UMAP (Becht et al., 2018; van der Maaten, 2014). Although the two representations are not identical, these clustering approaches converged on a number of interesting points. First, we observed C57-MSCs and BALB-MSCs cells distinctly clustered away from each other, supporting our previous observation that these cell types have distinct molecular profiles (Figure S11C). Second, we observed C57-MSCs clustered more tightly together when compared with BALB-MSCs, suggesting that the C57-MSCs are a more transcriptionally homogeneous population. These cell clustering approaches were supported by pairwise n-dimensional Euclidean distances between cells of the same sample and a shared nearest neighbor approach, which also demonstrated the BALB-MSC population was more transcriptionally heterogeneous than C57-MSCs (Figure S11D and S11E). Third, the overexpression of *Cd14* in BALB-MSCs homogenized their transcriptional profiles as observed by more unified clustering and smaller pairwise n-dimensional Euclidian distance measurements (Figures S11F and S11G). Interestingly, LPS exposure decreased Euclidean distances between cells of the same sample, suggesting that LPS-priming helped to homogenize transcriptional profiles in these cells. And finally, in the absence of LPS, BALB-MSC-CRISPRa-CD14 clustered away from BALB-MSC-CRISPRa-Scrambled, demonstrating that upregulation of the *Cd14* receptor shifts the ground state of these MSCs. To define these differences in ground state, we examined the differentially expressed genes between BALB-MSC-CRISPRa-Scrambled and BALB-MSC-CRISPRa-CD14 cells and characterized the functions of these genes using GO-term analysis (Figure 5E). Among the top GO-term categories enriched in BALB-MSC-CRISPRa-CD14 were several involved with immune function including “response to interleukin-4,” “killing cells of other organisms”, “neutrophil chemotaxis,” and “granulocyte chemotaxis”. In addition, we examined the biological processes enriched in BALB-MSC-CRISPRa-CD14 cells when compared with C57-MSCs-CRISPRa-Scrambled cells using GO-term analysis and identified the most enriched categories to be involved with “glycolytic process,” “ribosomal large subunit assembly,” and “neutrophil migration.” Together, these experiments demonstrate that the overexpression of CD14 in BALB-MSCs shifts their ground state toward one that is more amenable to elicit a rapid response to bacterial exposure.

## Discussion

In this work, we investigated the ability of MSCs from different genetic backgrounds to inhibit bacterial growth, examined molecular variations between MSCs, and utilized a systems level approach to identify genetic targets to improve antibacterial properties. We demonstrate that although MSCs from different genetic backgrounds may share similar cell surface markers, other phenotypes of these cells are not necessarily equivalent. This work highlights the phenotypic variation in MSCs and demonstrates a strategy to enhance desirable therapeutic properties in these cells using CRISPR tools.

Our initial observations were consistent with previous studies that demonstrated priming MSCs with bacteria or bacterial LPS could enhance antibacterial activity in these cells (Sung et al., 2016; Krasnodembskaya et al., 2010; Saeedi et al., 2019). However, we observed that the response to LPS stimulation in C57- and BALB-MSCs exhibited differential temporal dynamics and later found that these different LPS response rates may contribute to the difference in antibacterial activity between C57-MSCs and BALB-MSCs. Previous studies have demonstrated that mouse strain backgrounds, including C57BL/6 and BALB/c, can show dramatic differences in immune responses (Sellers et al., 2012; Watanabe et al., 2004). Through our RNA-seq and proteomics experiments, we identified that C57-MSCs and BALB-MSCs exhibited notable differences in the LPS signaling pathway. Of particular interest was the striking upregulation of the LPS receptor CD14 at the RNA and protein levels in C57-MSCs when compared with BALB-MSCs. CD14 was originally identified as a marker of monocytes and has since been shown to dramatically increase cell sensitivity to LPS (Gioannini et al., 2004; Perera et al., 1997; Goyert et al., 1986). CD14 is a co-receptor that binds LPS monomers and delivers them to the TLR4-MD-2 complex, initiating a signaling program that activates potent transcription factors, including NF-κB, AP-1, and IRFs, which regulate immune responses (Lu et al., 2008; Zanoni and Granucci, 2013; Pugin et al., 1994). We therefore tested whether upregulation of *Cd14* in BALB-MSCs could improve the rate of LPS response in these cells and enhance antibacterial activity, similar to that of C57-MSCs. Here, we utilized CRISPRa technology (SAM system) to upregulate endogenous *Cd14* gene expression as an approach to stably engineer MSCs with enhanced LPS recognition (Konermann et al., 2015). We observed BALB-MSCs and C57-MSCs overexpressing *Cd14* responded more quickly to LPS stimulation (as measured by NF-κB translocation) and displayed enhance antibacterial activity (as evident in inhibition of co-cultured *E. coli*). Surprisingly, we found that overexpression of *Tlr4* did not have the same effect. This suggests that *Cd14* may serve as a bottleneck in controlling sensitivity to LPS stimulation in MSCs, similar to what has been observed in other cell types (Perera et al., 1997; Gioannini et al., 2004). A recent study by Jiang et al. similarly concluded that *Cd14* deficiency in MSCs may be responsible for an attenuated LPS response (Jiang et al., 2019). Our work builds on the framework outlined by Jiang et al. and demonstrates that the overexpression of *Cd14* in MSCs accelerates LPS response through increased NF-κB nuclear translocation rate and subsequent changes in global gene expression. LPS priming through TLR4 activation has been utilized to control diverse therapeutic properties in MSCs, including (but not limited to) hematopoietic support (Ziegler et al., 2016; Wang et al., 2012; Shi et al., 2011), immunomodulation (Rashedi et al., 2017; Yan et al., 2014; Park et al., 2016; Waterman et al., 2010), and antibacterial activity (Saeedi et al., 2019; Sung et al., 2016). A recent report by Munir et al. demonstrated that LPS-primed MSCs have enhanced therapeutic properties, as administration of these cells dramatically accelerated wound healing by reshaping the wound site through increasing recruitment and activation of neutrophils and macrophages (Munir et al., 2020). How might combining *Cd14* overexpression in MSCs with TLR4-stimulation affect these diverse therapeutic properties? Subsequent studies in this field may benefit from the use of *Cd14* overexpression as a mechanism to reduce the amount of LPS required for stimulation or to amplify therapeutic effects. The role of CD14 in MSC biology has remained elusive because this protein, by early definitions, was considered a negative marker for this cell type (Dominici et al., 2006). However, studies have since identified MSC subpopulations in mice and humans that express the CD14 protein, further strengthening the conclusion that MSCs are a heterogeneous cell population even within a single organism (Jiang et al., 2019; Pilz et al., 2011).

The emergence of single-cell characterization technologies allows for a higher resolution molecular understanding of MSC subpopulations. These tools are beginning to be used to define MSC heterogeneity and to explore how genetic modifications and priming can impact MSC population diversity (Huang et al., 2019; Sun et al., 2020; Freeman et al., 2015; Gu et al., 2019). Strategies to limit heterogeneity and enrich homogeneous populations of MSCs are critical for ensuring safe and effective MSC-based therapies. We used 10X Genomics single-cell transcriptomics technology to determine the effect that *Cd14* expression and LPS priming have on MSC population heterogeneity. Interestingly, based on two methods of dimensionality reduction (tSNE and UMAP), C57-MSCs appear more homogeneous than BALB-MSCs, which may partially explain why the latter have a more attenuated LPS response. Unexpectedly, we observed that overexpression of CD14 in BALB-MSCs not only increased the speed with which the cells responded to LPS but also changed the transcriptional ground state of BALB-MSCs in the absence of LPS exposure. One possible explanation is that there are trace levels of LPS present in our culture system, not enough to stimulate wild-type BALB-MSCs but sufficient to activate signal transduction programs when recognition is facilitated by overexpression of *Cd14*. Indeed, there is evidence that CD14 can increase sensitivity to LPS in a number of physiological contexts (Haziot et al., 1996; Gioannini et al., 2004; Perera et al., 1997; Cao et al., 2009). Alternatively, CD14 may be recognizing a different ligand, such as Gram-positive cell wall components HSP60 or LAM, or other endogenous lipids present in culture (Wang et al., 1998; Kol et al., 2000; Landmann et al., 2000; Pugin et al., 1994; Yu et al., 1997). Regardless of the stimulating ligand, the expression of *Cd14* on the surface of MSCs makes them more responsive to LPS treatment, likely by transitioning these cells to a primed state.

Through our transcriptional profiling analyses, we observed that C57-MSCs and BALB-MSCs exhibited different expression of multiple TLRs. A notable example is that BALB-MSCs had higher baseline expression of *Tlr1*, *Tlr2*, and *Tlr6*, which encode proteins can form heterodimers (TLR1/2 and TLR2/6) to sense and respond to lipoprotein. In our LPS priming experiments, cells were treated with a standard LPS preparation (not an ultra-pure form) that includes remnants of other bacterial components including lipoproteins, allowing it to co-stimulate TLR2 in addition to TLR4. An elegant study by Kellogg et al. recently demonstrated that cells exhibit distinct NF-κB dynamics when stimulated by TLR2 versus TLR4 agonists (Kellogg et al., 2017). In addition, when cells are exposed to a mixture of TLR2 and TLR4 stimulating agents, single cells do not exhibit a hybrid response and instead respond to one ligand or the other (Kellogg et al., 2017). Kellogg et al. demonstrated that cells signaling through TLR4 exhibit a rapid and unified response to LPS stimulation, whereas cells exposed to TLR2 ligands exhibit a delayed and variable response between individual cells (Kellogg et al., 2017). Using NF-κB nuclear translocation assays in our study, we observed the majority of C57-MSCs responded quickly to LPS treatment, which is consistent with the TLR4 signaling dynamics reported by Kellogg et al. In contrast, when wild-type BALB-MSCs were treated with LPS they exhibited delayed and longer duration NF-κB translocation kinetics, which is more reminiscent of the TLR2 dynamics. It is therefore possible that in response to LPS treatment or bacterial exposure, C57-MSCs respond through a TLR4 signaling program, whereas BALB-MSCs respond through TLR2 signaling. Overexpression of CD14 in BALB-MSCs dramatically shifted their NF-κB nuclear translocation dynamics to a rapid response, similar to that of wild-type C57-MSCs. One interpretation of this result is that high levels of CD14 in MSCs increases their sensitivity to LPS treatment and promotes signaling through TLR4 rather than TLR2. This model is supported by the study from Sung et al. who demonstrated that TLR2 was not involved with antibacterial activity in MSCs, suggesting that TLR2-based signaling may be insufficient for direct inhibition of bacterial growth but may play a role in immunomodulation in response to infection (Sung et al., 2016). However, further experiments are needed to test this model by utilizing single-cell transcriptional profiles of CD14-expressing MSCs when exposed to TLR2 or TLR4 agonists.

A number of mechanisms may be at play when it comes to how MSCs limit bacterial growth. In previous studies, MSCs have been shown to exert antimicrobial pressure through secretion of bactericidal peptides LL-37 (Camp) and β-defensin 2 (Defb2) (Krasnodembskaya et al., 2010; Sung et al., 2016). However, we did not observe the MSCs used in this study to express Camp or Defb2 before or after LPS treatment, highlighting the phenotypic variation of different MSCs. There were, however, several notable genes with differential expression between MSC types and after LPS treatment that have established roles in limiting bacterial growth. In particular, lipocalin-2 (Lcn2) is a bacteriostatic peptide that inhibits bacterial iron-sequestering siderophores and has previously been demonstrated to protect mice from *E. coli*-induced pneumonia (Wu et al., 2010; Gupta et al., 2012). We observed an increase in Lcn2 expression after LPS treatment in both C57-MSCs and BALB-MSCs, and C57-MSCs showed robust expression of Lcn2 before LPS treatment as well. Another notable candidate is nitric oxide synthase 2 (Nos2), which was also expressed during routine growth in C57-MSCs and was observed to increase in expression in both MSC types following LPS treatment. Nitric oxide secretion is one mechanism by which immune cells can limit microbial growth, and MSCs can stimulate macrophages to increase NO production, which enhances their bacterial-killing properties (Bogdan et al., 2000; Kim et al., 2016a). To our knowledge, Nos2 expression in MSCs has not previously been implicated in their antibacterial activity. Finally, through scRNA-seq of CD14-upregulated MSCs we observed the enrichment of several GO-term categories involved with immune-related processes that may promote antibacterial activity in these cells (e.g. killing cells of other organisms and granulocyte chemotaxis). Although we did not identify the molecular mechanism directly inhibiting bacterial growth in this work, our molecular characterizations of these MSCs point to potential gene targets that could be modulated using gene-editing tools in subsequent antibacterial studies.

The work outlined in this study demonstrates another example of phenotypic diversity across MSCs. These phenotypic variations across MSCs are a challenge toward the development of safe, effective, and consistent MSC-based therapies. How can we ensure that MSCs isolated with distinct genetic backgrounds will exhibit the necessary therapeutic behaviors? This becomes increasingly problematic if autologous MSC transplants are used, requiring phenotypic characterization of each patient’s MSCs before administration. In addition, MSCs are inherently heterogeneous, even within populations that were isolated from a single source. How can we homogenize these cells and harness subpopulations that are enriched for desired properties? In this work we demonstrate that molecular priming and targeted genetic modifications using CRISPR tools can promote population homogeneity and drive cells toward desired phenotypic outputs. Although our studies demonstrate that CD14-expressing murine MSCs exhibit enhanced antibacterial properties *in vitro*, future studies involving *in vivo* mouse models of bacterial infection need to be performed to determine their potential clinical utility. In addition, to more fully understand the practical implications of this study toward the development of cell-based therapies in the clinic, the insights gained here will need to be investigated using human-derived MSCs from multiple donors and against additional bacterial pathogens. MSCs have immense therapeutic potential; however, their progression into the clinic has been stymied by our lack of understanding of these cells due to their inconsistencies and heterogeneity. As we continue to learn how these unique cells operate and develop methods that effectively direct their behavior, the closer we get toward safe and reliable MSC-based therapies.

### Limitations of the study

This study included several limitations that should be investigated in subsequent work to expand on the findings presented here. First, all experiments conducted in this study utilized commercially available bone-marrow-derived MSCs from mice. Follow-on studies using human MSCs would help to define the therapeutic implications of these findings. In addition, because these MSCs were obtained from a commercial vendor, the isolation and initial expansion of these cells was out of our control. In future studies, primary MSCs isolated from additional mouse strain backgrounds, different tissue types, and different human donors would help to further define the role of genetic background and source on antibacterial properties in MSCs. Third, this work did not include any *in vivo* experiments, so it is unclear how the administration of CD14-upregulated MSCs would impact resolution of microbial infection in animals. Finally, although we identified promising candidates through RNA-seq, we did not experimentally verify which molecules produced by the MSCs directly inhibited bacterial growth. Follow-up experiments should be conducted involving quantification of secreted proteins in the media and genetic dissection of pathways to identify the mechanism regulating antibacterial activity.

## STAR★Methods

### Key resources table

**Table:** 

REAGENT or RESOURCE	SOURCE	IDENTIFIER
**Antibodies**		

Mouse Mesenchymal Marker Antibody Panel	R&D Systems	SC018
Goat anti-rat IgG Alexa Flour 647 secondary antibody	Abcam	Abcam Cat# ab150159, RRID:AB_2566823
Goat anti-mouse IgG Alexa Fluor 488 secondary antibody	Abcam	Abcam Cat# ab150117, RRID:AB_2688012
NF-κB p65 (L8F6) Mouse mAb antibody	Cell Signaling	Cell Signaling Technology Cat# 6956, RRID:AB_10828935
PE anti-mouse CD14 antibody	BioLegend	BioLegend Cat# 150105, RRID:AB_2728188
APC anti-mouse TLR4 antibody	BioLegend	BioLegend Cat# 145405, RRID:AB_2562502

**Bacterial strains**		

*E. coli* strain K-12 MG1655	ATCC	700926

**Chemicals, peptides and recombinant proteins**		

LPS from *E. coli* 055:B5 standard	InvivoGen	Tlrl-b5lps
16% paraformaldehyde	Electron Microscopy Sciences	15710
Bovine Serum Albumin	Sigma-Aldrich	9048-46-8
0.1% Triton X-100	Thermo Fisher	BP151-100
DAPI	Invitrogen	D1306
TRIzol	Invitrogen	15596026
Lipofectamine 3000	Thermo Fisher	L3000015

**Critical commercial assays**		

LEGENDplex Mouse Inflammation Panel	Biolegend	740150
LEGENDplex Mouse Pro-inflammatory Panel	Biolegend	740007
PureLink RNA Mini Kit	Invitrogen/Thermo Fisher	12183020
RNA 6000 Nano Kit	Agilent	5067-1511
KAPA HyperPrep Kit with RiboErase (HMR)	Roche	KK8560
KAPA library quantification kit	Roche	KK4824
High Output v2.5 kit (150 cycles)	Illumina	20024907
MEM-PER Plus Membrane Protein Extraction Kit	Thermo Fisher	89842
Chromium Single Cell 3′ Reagent Kit v3	10X Genomics	CG000183
Agilent High Sensitivity DNA Kit	Agilent	5067-4627

**Deposited data**		

RNA-sequencing Data	NCBI Sequence Read Archive	NCBI SRA: PRJNA667557

**Experimental models: Cell lines**		

C57Bl/6 Mouse Mesenchymal Stem Cells	Cyagen	MUBMX-01001
Balb/c Mouse Mesenchymal Stem Cells	Cyagen	MUCMX-01001
C57Bl/6 Mouse Embryonic Fibroblasts	Blelloch lab, UCSF	N/A
NIH/3T3	ATCC	CRL-1658

**Oligonucleotides and plasmids**		

lenti-dCas9-VP64_Blast	Addgene	RRID:Addgene_61425
lenti-MS2-P65-HSF1_Hygro	Addgene	RRID:Addgene_61426
lenti-sgRNA(MS2)_zeo backbone	Addgene	RRID:Addgene_61427
Primers for CRISPRa sgRNAs, see Table S1	This paper	N/A
Lentiviral packaging vector psPAX2	Addgene	RRID:Addgene_12260
Lentiviral packaging vector pCMB-VSV-G	Addgene	RRID:Addgene_8454

**Software and algorithms**		

FlowJo	BD	V10
Prism	GraphPad	8
R	R	V3.6
HCS Studio Cell Analysis software	Thermo Fisher	2.0
bcl2fastq	Illumina	1.8
Fastp	https://github.com/OpenGene/fastp	NA
Kallisto	Pachter lab	0.46.0
DeSeq2	Bioconductor	1.22.2
Cell Ranger	10x Genomics	3.1

**Other**		

Mouse Mesenchymal Stem Cell Media	Cyagen	MUXMX-90011
Dulbecco’s Minimum Eagle Medium high glucose	Gibco	11965092
Fetal Bovine Serum (heat-inactivated)	Gibco	A3840001
Penicillin-streptomycin	Gibco	15140-122
Trypsin	Gibco	25200056
RPMI 1640 media	Gibco	1875093
MSC Adipogenic Differentiation Media	Cyagen	GUXMX-90031
MSC Osteogenic Differentiation Media	Cyagen	GUXMX-90021
Dulbecco’s Phosphate Buffer Saline	Gibco	14190144
EmbryoMax 0.1% gelatin solution	Sigma-Adrich	ES-006-B
Lysogeny Broth (LB)	Teknova	L9135
Lysogeny Broth (LB) - Agar	Teknova	L9110
Transwell plates	Thermo Fisher	140640

### Resource availability

#### Lead contact

Further information and requests should be directed to the lead contact, Raga Krishnakumar (rkrishn@sandia.gov).

#### Materials availability

Materials generated in this study, including plasmids and cell lines, can be requested by contacting the lead contact.

#### Data and code avalability

• RNA sequencing data was deposited into the National Center for Biotechnology Information Sequence Read Archive (https://www.ncbi.nlm.nih.gov/sra) and is available under the study accession NCBI SRA: PRJNA667557.
• This paper does not report original code.
• Any additional information required to reanalyze the data reported in this study is available from the lead contact upon request.

### Experimental model and subject details

#### Mammalian and bacterial cell culture

Bone marrow derived MSCs from 2-week-old male C57BL/6 and BALB/c mice were purchased commercially and routinely cultured using Mouse Mesenchymal Stem Cell Media (Cyagen US Inc.). Primary MEFs from C57BL/6 mice (Blelloch Lab, UCSF), primary MDFs from C57BL/6 mice (ScienCell Research Laboratories, Carlsbad, CA), NIH/3T3 cells from NIH Swiss mice (ATCC, Manassas, VA) and HEK 293T cells (ATCC) were routinely grown in high glucose, pyruvate DMEM (4.5 g/L D-glucose, 110 mg/L sodium pyruvate; Gibco^TM^ Thermo Fisher Scientific, Waltham, Massachusetts) supplemented with 10% heat inactivated FBS (Gibco^TM^ Thermo Fisher Scientific) and 1% penicillin-streptomycin. All mammalian cell culture was conducted in two-dimensional monolayers on tissue culture treated plastic dishes or well-plates in a humidified incubator at 37°C with 5% CO_2_. MSCs were used in experiments at a passage number ≤10 and subcultured when cells reached ∼80–90% confluence. CRISPR-modified MSCs were utilized at a passage number ≤15. All LPS-priming experiments were conducted in RPMI 1640 media (Gibco^TM^ Thermo Fisher Scientific) + 5% FBS without antibiotics using a standard preparation of LPS from *E. coli* 055:B5 (InvivoGen, San Diego, California) at a final concentration of 100 ng/mL. *E. coli* used in this study were strain K-12 MG1655 (ATCC) and were routinely cultured in LB media (Teknova, Hollister, California).

### Methods details

#### Differentiation assays

Differentiation assays were performed using adipogenic and osteogenic differentiation media as per instructions provided (Cyagen Biosciences, Inc.). In brief, for adipogenic assays, cells were plated at 2 × 10^4^ cells/cm^2^ in a 6-well tissue culture treated dish in MSC basal media and grown until cells reached 100% confluence. Cells were then induced to undergo adipogenesis by culturing cells for 3 days in MSC Adipogenic Differentiation Induction Media (Cyagen Biosciences, Inc.), followed by 1 day in MSC Adipogenic Differentiation Maintenance Media (Cyagen Biosciences, Inc.). The process of cycling adipogenic induction and maintenance medias was repeated for a total of three times. Cells were then fixed using 4% paraformaldehyde in PBS for 30 minutes at room temperature, washed twice with PBS and stored at 4 °C. Fixed cells were then stained with an Oil Red O solution (diluted 3:2 in DI water and filtered through filter paper) for 30 minutes and washed 3 times with PBS. For osteogenic differentiation assays, 6-well cell culture dishes were first coated with 0.1% gelatin solution for 30 minutes at room temperature and then gelatin solution was removed. Cells were seeded at 2 × 10^4^ cells/cm^2^ in MSC basal media and grown to ∼70% confluence. Cells were then put in MSC Osteogenic Differentiation Media (Cyagen Biosciences, Inc.), and this media was replaced every 3 days for a total incubation of 2 weeks. After two weeks incubation in Osteogenic Differentiation Media, cells were fixed with 4% paraformaldehyde in PBS as described above. Fixed cells were then stained with 1 mL Alizarin Red S working solution (Cyagen Biosciences, Inc.) for 5 minutes at room temperature, and then washed 3 times with PBS. Oil red O and Alizarin Red S stained cells were imaged using phase contrast microscopy with an EVOS XL Core Cell Imaging System (Thermo Fisher Scientific).

#### Immunostaining MSC surface markers and flow cytometry

Cells were fixed with 4% paraformaldehyde in PBS for 15 minutes at room temperature and then washed twice with PBS. Fixed cells were incubated in Blocking Buffer (3% BSA in PBS, filtered with 0.22 μm filter) at room temperature overnight. For mouse MSC surface markers, cells were stained using the Mouse Mesenchymal Marker Antibody Panel (R&D Systems). Primary antibodies were added to cells at a concentration of 1 μg/mL diluted in Blocking Buffer and incubated at room temperature for 1 hour on a rocker. Cells were then washed twice with PBS and resuspended in goat anti-rat IgG Alexa Flour 647 secondary antibody (Abcam, Cambridge, United Kingdom) diluted 1:1000 in Blocking Buffer and incubated at room temperature for 30 minutes. Cells were then washed and resuspended in PBS, and fluorescence levels of 10,000 cells were measured using a BD Accuri C6 Plus flow cytometer. Flow cytometry data was analyzed and plotted using FlowJo v10 (BD, Franklin Lakes, NJ).

#### Antibacterial assays

Protocols for antibacterial assays used in this study were adapted from previously described methods (Krasnodembskaya et al., 2010). Here, MSCs were trypsinized, quenched with media, and then washed twice with PBS. Cells were then resuspended in RPMI +5% FBS without antibiotics and cell density was quantified using a Bio-Rad TC20 automated cell counter. Cells were added to tissue-culture treated multi-well dishes at a concentration of 1.25 × 10^5^ cells/cm^2^ in RPMI +5% FBS without antibiotics (+/− LPS 100 ng/mL) and incubated overnight at 37 °C + 5% CO_2_. *E. coli* strain K-12 MG1655 was grown overnight in LB liquid at 37 °C while shaking at ∼220 RPM. The following day, *E. coli* cells were enumerated by OD_600_ measurement (1 OD = 4 × 10^8^ CFU/mL). Bacterial cells were then diluted in RPMI +5% FBS and added directly to mammalian cell culture wells to a final concentration of 1 × 10^3^ CFU/mL (final volume of *E. coli* suspension added was ∼2% of total incubation volume). Mammalian cells and *E. coli* were co-incubated at 37 °C + 5% CO_2_ for 6 hours without agitation. After 6 hours, *E. coli* viability was measured by plating dilutions of bacterial cell suspensions onto LB agar and counting CFUs. Transwell antibacterial assays were performed using the same protocol as above, except bacteria were inoculated into the upper chamber of a permeable transwell insert containing a 0.4 μm polycarbonate membrane. Statistical significance between groups was determined using t-test and plotted using Prism 8 software (GraphPad). For antibacterial assays, we used the pwr.t.test function in R to calculate that given the CFU values we were obtaining to have an average power of 80% across conditions we needed n = 5 per condition (some conditions have more). In addition, we used the Benjamini-Hochberg procedure which adjusted for multiple comparisons to determine that all p values below 0.044 represent significant results.

#### NF-κB nuclear translocation assays

MSCs were plated into black-walled 96-well glass bottom tissue culture plates in RPMI media + 5% FBS at a cell density of ∼60,000 cells / cm2. Adherent cells were left untreated, or treated with LPS from E. coli 055:B5 (100 ng/mL) and collected at specific timepoints post-exposure (0.083 hr, 0.25 hr, 0.5 hr, 1 hr, 2 hrs, and 18 hrs). Media was removed from wells and cells were fixed with 4% paraformaldehyde in PBS for 15 minutes at room temperature, and then washed twice with PBS. The cells were then incubated in Blocking Buffer B (3% BSA, 3% normal goat serum, 0.1% Triton X-100 in PBS, filtered through 0.22 μm filter) for 1 hour at room temperature. NF-κB p65 (L8F6) Mouse mAb antibodies (Cell Signalling Technology, Danvers, MA) diluted in blocking buffer were then added to the fixed cells and incubated for 18 hours at 4°C. The samples were then washed 3 times with 0.1% Triton X-100 in PBS before incubation with goat anti-mouse IgG Alexa Fluor 488 secondary antibody (Abcam) for 1 hour at room temperature. The cells were then washed once with PBS and incubated with DAPI diluted in PBS for 10 minutes, followed by two more PBS washes.

Images of the cells were acquired using the CellInsight CX7 High-Content Screening platform and quantified using the HCS Studio Cell Analysis software (Thermo Fisher Scientific). Background subtraction was first performed, followed by image segmentation using the DAPI channel to count the total number of cells imaged in each field of view and record the average intensity values of the p65 channel for each nucleus. A ring region of interest (ROI) surrounding the nucleus for each cell was also created to measure antibody fluorescence in the cytoplasm. NF-κB p65 nuclear translocation was identified by counting cells yielding a ratio of nuclear average intensity to cytoplasmic intensity greater than 1. For nuclear translocation assays, we used the pwr.t.test function in R to calculate that given percent translocation values we were obtaining across time points, to have an average power of 80% across comparisons we needed n = 3 per condition. In addition, we used the Benjamini-Hochberg procedure which adjusted for multiple comparisons to determine that all p values below 0.00013716 represent significant results.

#### Cytokine profiling

For cytokine analyses, ∼250,000 cells were plated in 24-well tissue culture treated dishes and grown in 0.5 mL RPMI +5% FBS and incubated at 37°C + 5% CO2 overnight. Cells were either left untreated, or treated with LPS from *E. coli* 055:B5 at a concentration of 100 ng/mL for ∼18 hrs. Conditioned media was collected and centrifuged briefly to remove debris. Bead-based immunoassays were performed using LEGENDplex assays with the pre-defined Mouse Inflammation Panel and the Mouse Proinflammatory Chemokine Panel. LEGENDplex immunoassays were performed as described by the manufacturer’s protocols (BioLegend, San Diego, CA). LegendPlex assays were measured using a BD Accuri C6 Plus flow cytometer, and data analyzed using LEGENDplex software (BioLegend) and Prism 8 (GraphPad Software, San Diego, CA).

#### Population level RNA-seq and data analysis

To harvest RNA from cells for RNA-seq, cells were resuspended in TRIzol reagent (Invitrogen^TM^, Thermo Fisher Scientific) and stored at −80°C until further processing (n = 3 biological replicates per condition). For RNA-seq of cells during standard growth, RNA was collected from cells when they reached ∼80–90% confluence. For the LPS-timecourse RNA-seq experiment, cells were plated at a concentration of 125,000 cells/cm^2^, treated with LPS (100 ng/mL) or without (samples labeled as 0 hr timepoint), and collected in TRIzol at 0.5 hr, 2 hr, and 18 hr post-exposure. RNA was purified from TRIzol using PureLink RNA Mini Kit (Invitrogen^TM^, Thermo Fisher Scientific) and RNA concentration was measured with a Qubit fluorometer (Thermo Fisher Scientific). The quality of RNA preps was determined using the RNA 6000 Nano Kit on a Bioanalyzer (Agilent Technologies, Santa Clara, CA) and all samples used in this study had RNA integrity numbers >7. RNA-seq libraries were generated using the KAPA HyperPrep Kit with RiboErase (HMR) and KAPA dual-indexed adapters (Roche Sequencing and Life Sciences, Wilmington, MA). RNA-seq libraries were quantified using the High Sensitivity DNA Chip on a Bioanalyzer (Agilent Technologies, Santa Clara, CA) and KAPA library quantification kit (Roche). RNA-seq libraries were sequenced using the Illumina NextSeq 500/550 platform with the High Output v2 kit (150 cycles) on paired-end mode (Illumina Inc, San Diego, CA). BCL files were converted to FASTQ and demultiplexed using the bcl2fastq conversion software (Illumina, Inc.). Quality filtering and adaptor trimming were performed using fastp (Chen et al., 2018) with following parameters: --qualified_quality_phred 25, --cut_window_size 5 -3 and --cut_mean_quality 25. Kallisto was used for alignment-free mapping with 100 bootstraps per sample to calculate transcript counts (Bray et al., 2016). PCA was performed using the prcomp function in R, and MDS performed with edgeR (Robinson et al., 2010). Differential expression analysis was performed using DeSeq2 (Love et al., 2014) with significant genes being defined with a fold change >2 and an adjusted p value < 0.05. Pathway analysis was performed and visualized using Pathview (Luo and Brouwer, 2013). GO-term analysis was performed using PANTHER (Mi et al., 2019; Ashburner et al., 2000) and visualizations were plotted in R.

#### Quantitative membrane proteomics

Cells were prepared using standard growth conditions as described above and processed when cell density reached ∼75–95% confluency. Adherent cells were washed twice with DPBS and detached from the surface of the plate using a cell scraper into centrifuge tubes. Cells were centrifuged for 5 minutes at 300 RCF, supernatant removed, and cell pellets were flash frozen in liquid nitrogen. Each cell type was collected in biological triplicate and sent for membrane processing, protein extraction and LC-MS/MS (performed by MS Bioworks, Ann Arbor, Michigan). Cell pellets were processed using the Pierce MEM-PER reagent according to the manufacturers protocol. Extracted proteins were concentrated by trichloroacetic acid, and protein pellets were washed with ice cold acetone and solubilized in 8M Urea, 150mM NaCl, 50mM Tris-HCl pH8, 1X Roche Complete protease inhibitor. The protein concentration of the extract was determined by Qubit fluorometry. 25μg of protein was reduced with dithiothreitol, alkylated with iodoacetamide and digested overnight with trypsin (Promega). The digestion was terminated with formic acid and desalted using an Empore SD solid phase extraction plate. 2μg of each peptide sample was analyzed by nano LC-MS/MS with a Waters NanoAcquity HPLC system interfaced to a ThermoFisher Q Exactive. Peptides were loaded on a trapping column and eluted over a 75μm analytical column at 350 nL/min; both columns were packed with Luna C18 resin (Phenomenex). The mass spectrometer was operated in data-dependent mode, with the Orbitrap operating at 70,000 FWHM and 17,500 FWHM for MS and MS/MS respectively. The fifteen most abundant ions were selected for MS/MS. 4hrs of instrument time was used for the analysis of each digest. Data were processed with MaxQuant version 1.6.0.13 (Tyanova et al., 2016). Differential protein expression analysis and clustering was performed using the DEP R package (Zhang et al., 2018) to identify and characterize protein composition of cell membranes. GO-term analysis of membrane proteins was performed using PANTHER (Mi et al., 2019).

#### CRISPR-mediated gene activation in MSCs

Gene activation by CRISPR was performed using the CRISPR/Cas9 Synergistic Activation Mediator (SAM) system (Konermann et al., 2015). C57-MSC-CRISPRa and BALB-MSC-CRISPRa were constructed through lentiviral transduction of vectors lenti-dCas9-VP64_Blast (Addgene # 61425) and lenti-MS2-P65-HSF1_Hygro (Addgene #61426). Mouse CD14 and TLR4 sgRNAs sequences were designed using the sgRNA design tool from the Zhang Lab (http://sam.genome-engineering.org/database/). Six sgRNAs were designed per gene with different distances upstream of the transcription start site. Primers for individual sgRNAs were annealed and cloned into the lenti-sgRNA(MS2)_zeo backbone (Addgene #61427) using the Golden Gate cloning reaction into the BsmBI site (For primers sequences see Key resources table). sgRNA plasmids were checked for correct sequence integration using Sanger Sequencing (Genewiz Inc, South Plainfield, NJ). Each sgRNA lentiviral construct was transfected into HEK 293T cells along with packaging vectors psPAX2 (Addgene #12260) and pCMV-VSV-G (Addgene #8454) using Lipofectamine 3,000 (Thermo Fisher Scientific), and incubated for 48 hours. HEK 293T media containing lentivirus was then filtered through a 0.45 μm syringe filter, added to MSCs with polybrene (10 μg/mL), and incubated for 24 hours. Following 24-hour transduction, the media was replaced with fresh media with appropriate antibiotic selection. Following selection, expression levels of CD14 and TLR4 were tested in CRISPRa cells expressing different sgRNAs using immunostaining followed by flow cytometry. Cells were live-stained using PE anti-mouse CD14 antibody or APC anti-mouse TLR4 antibody (BioLegend), then fixed with 4% paraformaldehyde for 15 minutes, washed and diluted in PBS. Stained cells were then examined using a BD FACSMelody^TM^ Cell Sorter (BD Biosciences) and data was analyzed using FlowJo v10.

#### Single-cell RNA-seq sample preparation

The cells used in scRNA-seq studies were C57-MSC-CRISPRa-Scrambled (expressing scrambled sgRNA-1), BALB-MSC-CRISPRa-Scrambled (expressing scrambled sgRNA-1), and BALB-MSC-CRISPRa-CD14 (expressing CD14-83 sgRNA). All cells were plated at a density of 125,000 cells/cm in 24-well plates in RPMI +5% FBS and either treated with LPS (100 ng/mL for 0.5 hr, 2 hr, and 18 hr) or left untreated. Cells were then trypsinized, washed with PBS, and resuspended in RPMI +5% FBS at a final concentration of 1 × 10^6^ cells/ml. Cell suspensions were then processed to isolate ∼2,000 individual cells per sample using the Chromium Single Cell 3′ Reagent Kit v3 and 10x Chromium Controller following protocols as outlined in the corresponding 10x user guide Rev. B (10x Genomics, Pleasanton, CA). All subsequent steps (reverse transcription, cDNA amplification, and gene expression library construction) were performed as described in the 10x Chromium Single Cell 3′ Reagent Kits v3 Rev B User Guide (10x Genomics). Final libraries were quantified using a Qubit Fluorometer with hsDNA reagent (Thermo Fisher Scientific) and run on a Bioanalyzer High Sensitivity DNA chip (Agilent Technologies). Libraries were sequenced using an Illumina NextSeq 500/550 platform with the High Output v2.5 kit with 26 × 98 paired-end reads. Sequencing output was processed using Cell Ranger software protocols (10x Genomics). In brief, BCL files were demultiplexed and converted to fastq using Cell Ranger mkfastq. Fastq files were processed for alignment, filtering, barcode counting, and UMI counting using the Cell Ranger “count” feature, and pooled by each GEM using the Cell Ranger aggr pipeline.

#### Single-cell RNA-seq analysis

Seurat and Monocle3 R packages were used to process, filter and normalize single-cell RNA-seq data (Trapnell et al., 2014; Cao et al., 2019; Qiu et al., 2017a; Becht et al., 2018; Stuart et al., 2019; Butler et al., 2018), and figures for tSNE, U-MAP and violin plots were generated by modifying standard pipelines. Briefly, samples were adjusted for cell cycle stage and filtered as follows: number of Features above 200, and percentage RPS and RPL <12. Samples were then log-normalized and scaled. Data processed across two different experimental runs was normalized using COMBAT (Johnson et al., 2007; Tran et al., 2020). The UMAP settings were reduction = "pca", dims = 1:10, n.neighbors = 10, n.components = 10, min.dist = 0.05, local connectivity = 5. The tSNE settings were dims = 1:10, dims.use = 1:10, reduction.use = "pca". All code provided upon request. Euclidian distance between points was calculated using 10 dimensions (for both tSNE and UMAP). Differentially expressed genes were identified using the FindMarkers module in Seurat. Gene ontology analysis on differentially expressed genes was performed using PANTHER (Mi et al., 2019).

### Quantification and statistical analysis

Statistical analyses for experiments were performed using Microsoft Excel, GraphPad Prism, and the R statistical computing language. The details for statistical analyses for each experimental procedure are described in the appropriate methods sections and corresponding figure legends.

## Supplemental information

Document S1. Figures S1–S11 and Table S1

DataS1. Antibacterial Assay Data, related to Figure 1 and Figure 5

DataS2. Cytokine Profiling Data, related to Figure 1

DataS3. NF-κB Nuclear Translocation Data, related to Figure 2 and Figure 5

DataS4. Quantitative Membrane Proteomics Raw Data, related to Figure 4
