# Supplementary material for: Upregulation of CD14 in mesenchymal stromal cells accelerates lipopolysaccharide-induced response and enhances antibacterial properties
Source: iScience. 2022 Jan 12;25(2):103759. doi: 10.1016/j.isci.2022.103759 (PMC8814754; doi:10.1016/j.isci.2022.103759)
Supplement: Document S1. Figures S1–S11 and Table S1 [file mmc1.pdf]

iScience, Volume *25*

Supplemental information

Upregulation of CD14 in mesenchymal stromal cells accelerates lipopolysaccharide-induced response and enhances antibacterial properties

Matthew P. Hirakawa, Nikki Tjahjono, Yooli K. Light, Aleyna N. Celebi, Nisa N. Celebi, Prem Chintalapudi, Kimberly S. Butler, Steven S. Branda, and Raga Krishnakumar

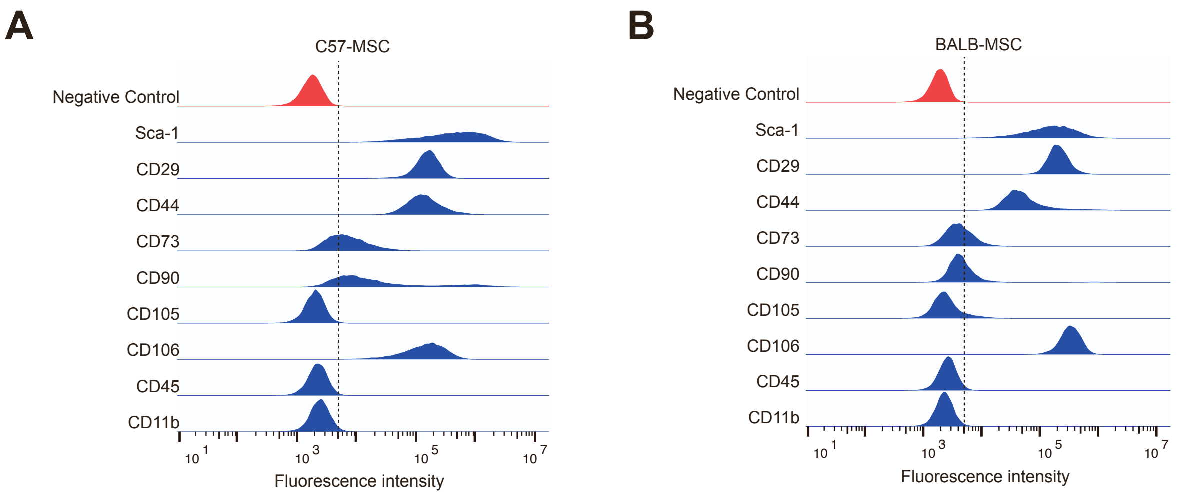

Figure S1. Analysis of traditional MSC cell-surface markers in different MSC types, related to Figure 1. Cells were inspected for the expression of MSC cell-surface markers by immunostaining with the Mouse Mesenchymal Marker Antibody Panel (R&D Systems) followed by flow cytometry. Both **(A)** C57-MSCs and **(B)** BALB-MSCs stained positively (>99% of cells) for the MSC markers Sca-1, CD29, CD44 and CD106. The MSC markers CD73 and CD90 were observed only in subsets of cells analyzed indicating population heterogeneity that has been observed with these proteins in MSCs. C57-MSCs and BALB-MSCs exhibited negative or low staining of CD105, respectively, and subpopulations of CD105-negative MSCs have also been previously characterized. The negative MSC markers, CD45 and CD11b, were not detected in either C57-MSC or BALB-MSCs. Plots shown are one representative of three independent experiments.

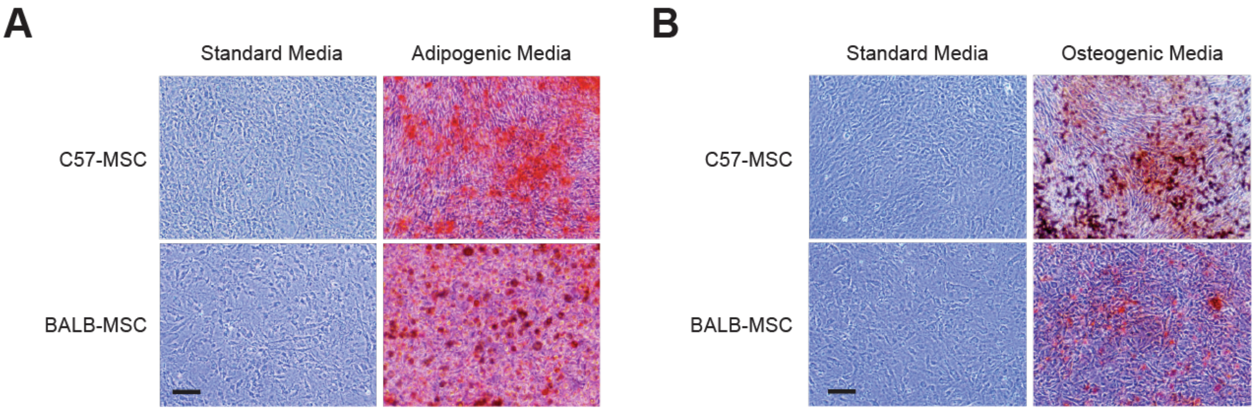

Figure S2. MSC differentiation assays, related to Figure 1. C57-MSCs and BALB-MSCs were tested for their ability to differentiate using **(A)** adipogenic and **(B)** osteogenic assays (Cyagen, Inc). Using this approach, we observed both cell types to be capable of adipogenesis and osteogenesis as visualized by Oil Red O and Alizarin Red S staining, respectively (Scale bars = 200 um). Images shown are representative of at least 3 independent experiments.

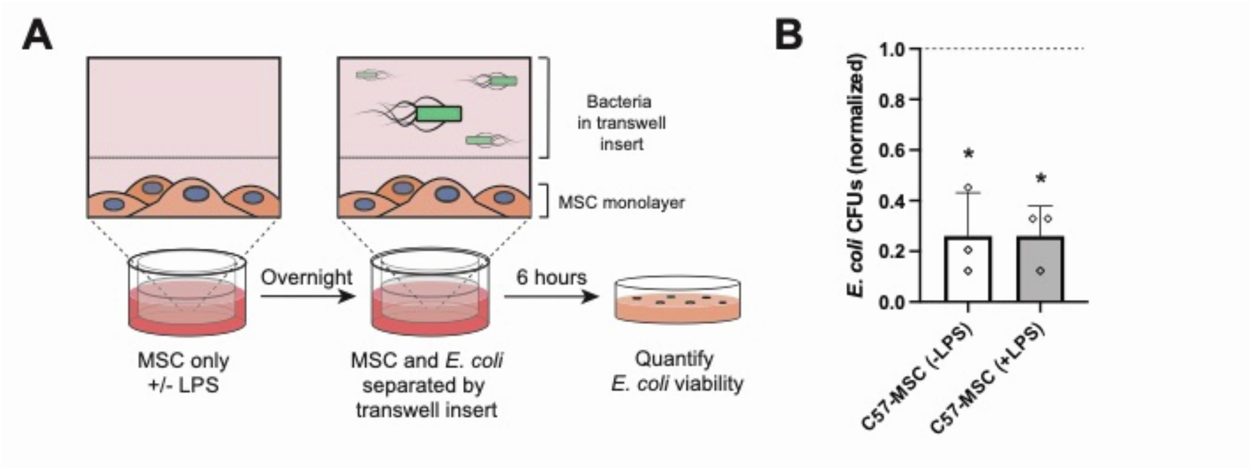

Figure S3. Antibacterial assays with physical separation of MSCs and *E. coli*, related to Figure 1. To test if MSCs needed to be in direct contact with bacteria to inhibit bacterial growth, antibacterial assays were performed using transwell inserts to physically separate *E. coli* from MSCs. **(A)** These experiments were performed like those in Figure 1, however *E. coli* were added to the upper chamber of a transwell insert so they could not directly contact MSCs in the bottom section of the well. **(B)** *E. coli* abundance was quantified using CFU assays after 6-hrs incubation in the transwell inserts, and normalized to CFU values of *E. coli* controls grown in identical transwell systems but in monoculture. Here, we observed that MSCs inhibited *E. coli* growth with and without LPS-priming when compared to a control with media only ($p = 0.03$ and $p = 0.04$, respectively). Bars depict the mean with error bars representing SD, statistical significance was determined using t-test on three biological replicates, and * represents $p < 0.05$.

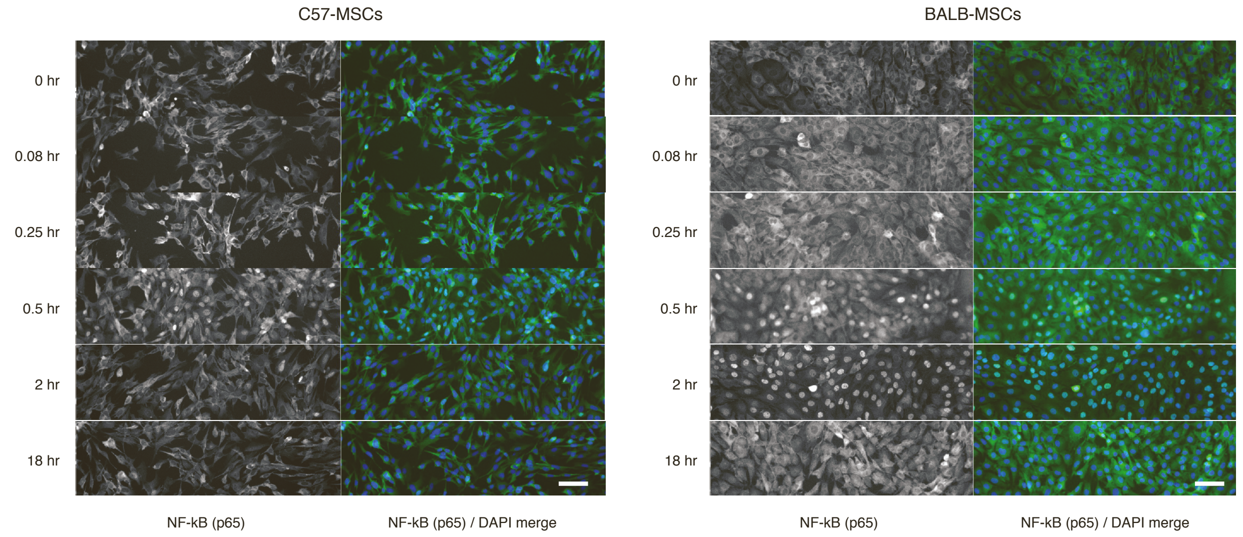

Figure S4. Time-course microscopy of NF-κB nuclear translocation in MSCs after LPS stimulation, related to Figure 2. Representative images depicting NF-κB localization in C57-MSCs (left) and BALB-MSCs (right) at six time points after LPS exposure (0.083 hr, 0.25 hr, 0.5 hr, 1 hr, 2 hrs, and 18 hrs). To visualize NF-κB localization, MSCs were fixed with paraformaldehyde and stained using an NF-κB p65 (L8F6) mouse primary antibody and Alexa Flour 488 secondary antibody. To visualize MSC nuceli, the cells were stained with DAPI. Cells were imaged and analyzed using a CellInsight CX7 High-Content Imager (scale bars = 50 um). The maximum nuclear localization of NF-κB was observed at 0.5 hrs in C57-MSCs and 2 hrs in BALB-MSCs (see Figure 2A for quantification data, and the materials and methods section for more details on the imaging and quantification procedure).

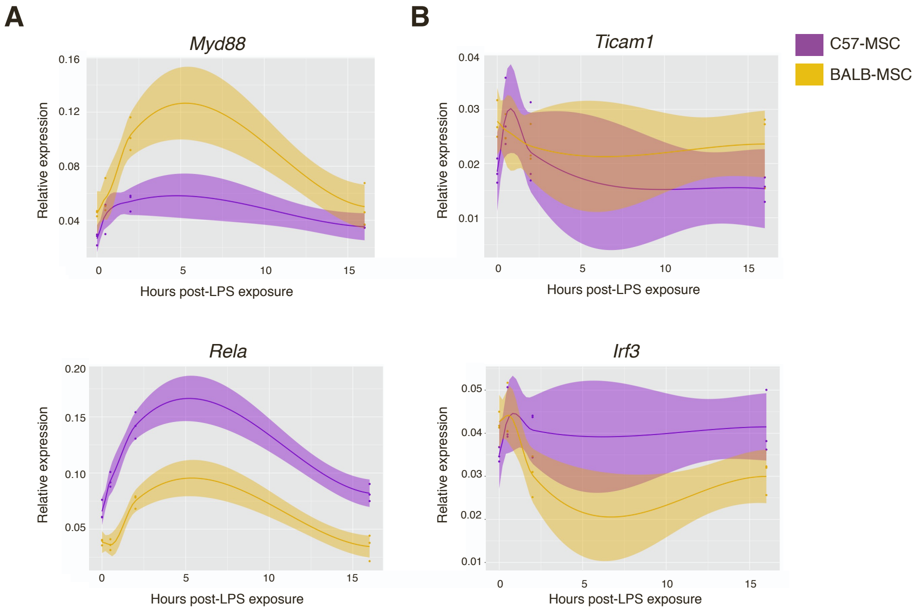

Figure S5. Gene expression changes of TLR4-adapter genes and target transcription factors in response to LPS, related to Figure 2. To serve as an internal control for gene induction in response to LPS, the relative expression levels (FPKM) of genes involved with the **(A)** MYD88-dependent and **(B)** MYD88-independent TLR4 signaling pathways were examined at different timepoints using RNA-seq. At 2-hrs post LPS exposure, we observed significant upregulation of *Myd88* in C57-MSCs and BALB-MSCs compared to unstimulated cells (padj = 1.05E-06 and 3.97E-18, respectively). Similarly, we observed significant upregulation of *Rela* in both C57-MSCs and BALB-MSCs compared to unstimulated cells (padj = 6.39E-13 and 2.16E-17, respectively). Alternatively, we did not observe upregulation of *Ticam1* or *Irf3* in response to LPS in either C57-MSCs or BALB-MSCs suggesting these cells may be responding to LPS through a MYD88-dependent pathway.

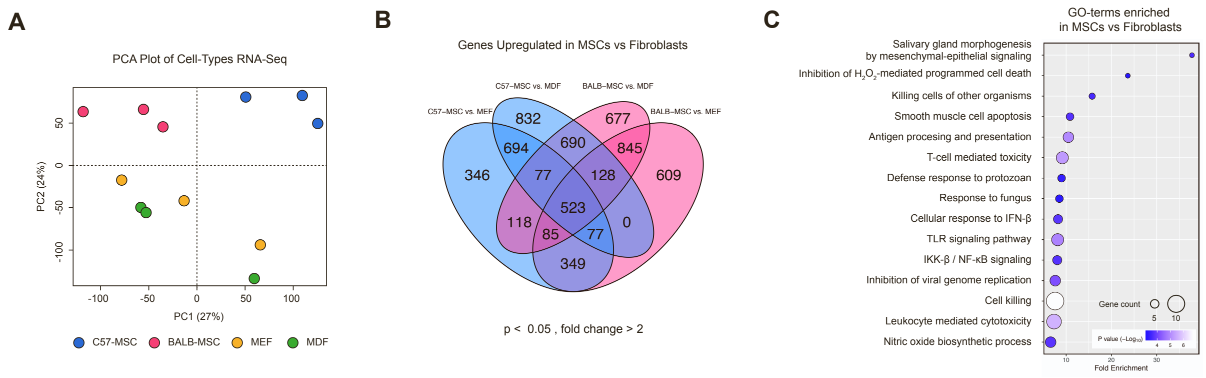

Figure S6. Transcriptional differences between MSCs and fibroblasts, related to Figure 3. **(A)** PCA plot depicting transcriptional profiles using RNA-seq of C57-MSCs, BALB-MSCs, MEFs and MDFs under standard growth conditions. **(B)** Venn diagram showing numbers of genes upregulated in pairwise comparisons of MSCs vs fibroblasts (upregulated genes were defined as having a fold change > 2 and an adjusted P < 0.05). The center overlapping area of 523 genes represented shared upregulated genes between both MSC types when compared to both fibroblasts. **(C)** These MSC-specific genes were then inspected for functional enrichment using GO-term analysis. The most functionally enriched GO category involved the regulation of branching involved in salivary gland morphogenesis by mesenchymal-epithelial signaling (GO:0060665), which was a result of upregulation of Fgf7, Hgf, and Met. Of particular note, MSCs were enriched for the TLR-signaling pathway (GO:0002224) that included higher expression of genes involved with responding to bacterial PAMPs including Lbp, Tlr2 and Irf1. Additionally, MSCs were enriched for the genes involved with cell killing (GO:0031341) which included chemokines (Cxcl1 and Cxcl5), MHC genes (H2-BI, H2-T22, H2-Q6, H2-Q2, H2-K1), as well as pro-apoptotic genes (Stat5a, Bcl2I11, Gapdh, Arrb2).

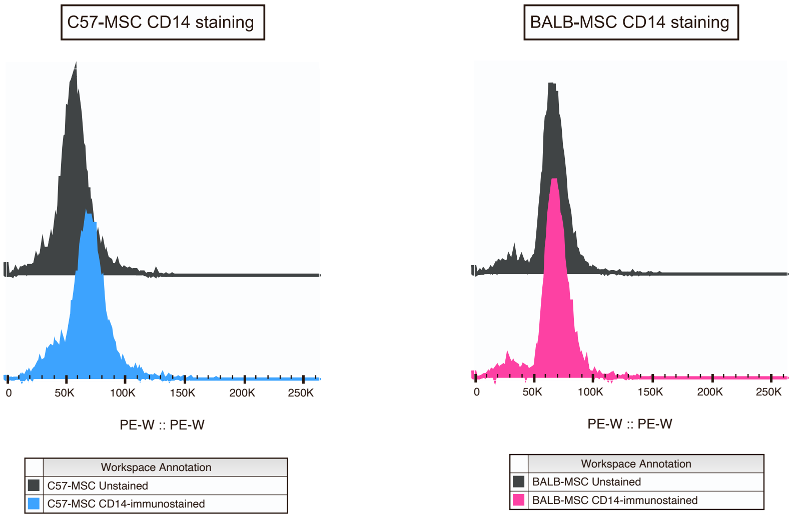

Figure S7. CD14 immunostaining in C57-MSCs and BALB-MSCs, related to Figure 4. Both MSC types were live-stained using a PE conjugated anti-mouse CD14 antibody and expression levels were measured using flow cytometry. The mean fluorescence intensity (MFI) of immunostained MSCs was compared to MFIs from respective unstained controls. Using this approach, we observed increased fluorescence intensity of C57-MSCs stained with CD14 when compared to their unstained controls (MFI = 68198 vs. 57669, respectively), indicating the presence of CD14 protein expression. Alternatively, the BALB-MSCs stained with CD14 were more similar to their unstained controls (MFI = 67100 vs. 65679, respectively), indicating these cells have extremely low or absent CD14 expression. Results shown are one representative of three independent experiments.

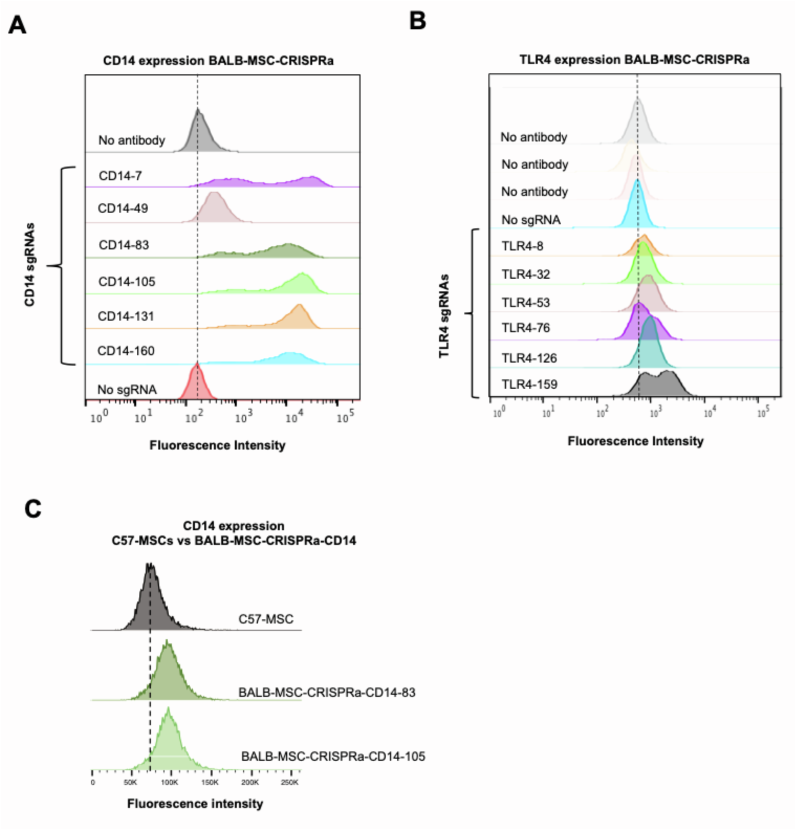

Figure S8. Comparison of sgRNAs to activate protein expression in BALB-MSC-CRISPRa cells, related to Figure 5. BALB-MSCs expressing the CRISPRa SAM system were transduced with lentiviral constructs to express one of six sgRNAs that target either **(A)** CD14 of **(B)** TLR4. Each sgRNA was designed to target a different distance upstream of the transcription start site (represented by the number after the gene name). MSCs were live-stained using anti-CD14-PE or anti-TLR4-APC antibodies and examined for fluorescence intensity using flow cytometry. Negative controls used here were BALB-MSC-CRISPRa not expressing sgRNA with no antibody staining (labeled no antibody), or the same cells treated with antibody (labeled no sgRNA). **(C)** The two sgRNAs that were selected for subsequent experiments were additionally tested for CD14 expression levels when directly compared to wildtype C57-MSCs. In these experiments, we observed the BALB-MSC-CRISPRa-CD14 cells to exhibit slightly elevated CD14 expression compared

to C57-MSCs. Plots shown are representative staining of one of three independent experiments.

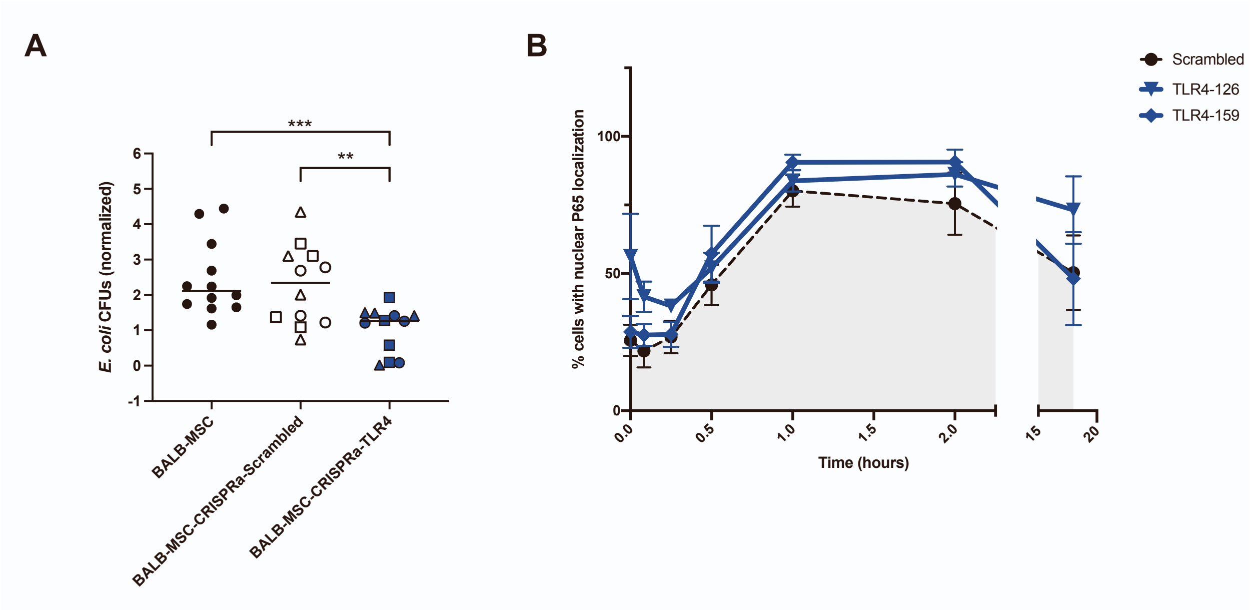

Figure S9. Functional analysis of TLR4 overexpression in BALB-MSCs, related to Figure 5. (A) BALB-MSCs overexpressing TLR4 were examined for their antibacterial properties by co-culturing these MSCs with *E. coli* for 6 hours and measuring the CFUs in the media (For scrambled sgRNAs: circle = non-targeting_control_1, square = non-targeting_control_2, triangle = non-targeting_control_3; For TLR4 sgRNAs: circle = TLR4-53, square = TLR4-126, triangle = TLR4-159). CFUs were normalized to a corresponding *E. coli* monoculture control performed alongside each biological replicate, and statistical significance was determined using t-test (Lines reference the median, and statistical significance is represented by ** P < 0.01, *** P<0.001). **(B)** BALB-MSC-CRISPRa-TLR4 cells were also inspected for their rates of NF-κB nuclear translocation after LPS-exposure compared to cells expressing a non-targeting "Scrambled" control. Overexpression of *Tlr4* did not lead to a change in NF-κB nuclear translocation rates in these cells. Data points represent the mean of at least 3 biological replicates, and error bars represent SEM.

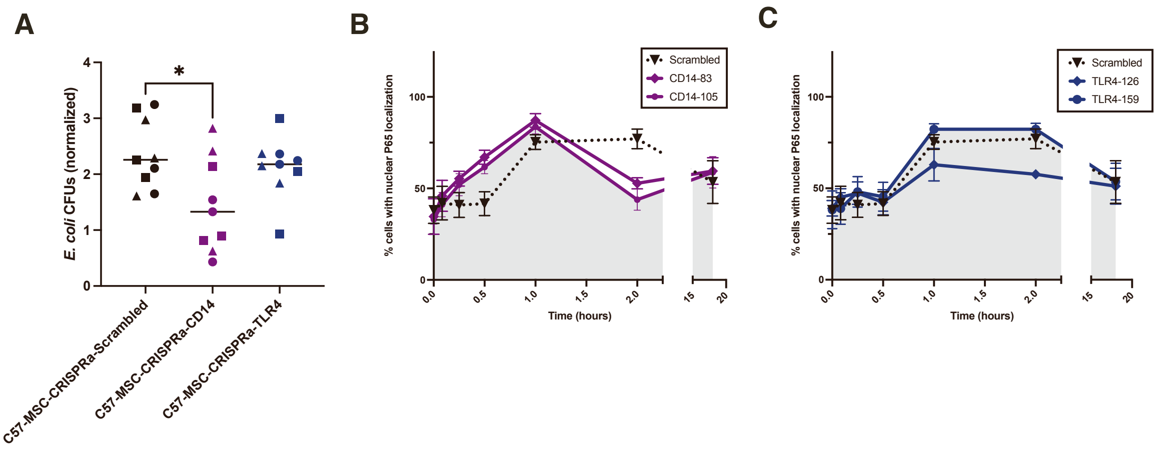

Figure S10. Antibacterial activity and LPS-response kinetics in C57-MSCs with upregulated *Cd14* or *Tlr4,* related to Figure 5. **(A)** C57-MSCs overexpressing *Cd14* or *Tlr4* via CRISPRa SAM system were examined for antibacterial properties by co-culturing with *E. coli* for 6 hours and measuring the CFUs in the media (For scrambled sgRNAs: circle = non-targeting sgRNA 1, square = non-targeting sgRNA 2, triangle = non-targeting sgRNA 3; For CD14 sgRNAs: circle = CD14-83, square = CD14-105, triangle = CD14-131; For TLR4 sgRNAs: circle = TLR4-53, square = TLR4-126, triangle = TLR4-159). CFUs were normalized to a corresponding 'media only' control performed alongside each biological replicate (Lines reference the median, and statistical significance is represented by *, $p < 0.05$). **(B)** NF-κB nuclear translocation assays in C57-MSC-CRISPRa-CD14 cells after LPS-exposure. We observed a significant increase in nuclear NF-κB at 2-hrs post LPS-exposure in C57-MSC-CRISPRa-CD14 when compared to cells expressing a non-targeting scrambled sgRNA (Data points represent mean with error bars +/- SEM, n = 3 biological replicates, $p < 0.05$). **(C)** NF-κB nuclear translocation assays were also performed in C57-MSC-CRISPRa-TLR4, however there was no significant difference

observed when these cells were compared to cells expressing a scrambled control (Data points represent mean with error bars +/- SEM, n = 3 biological replicates).

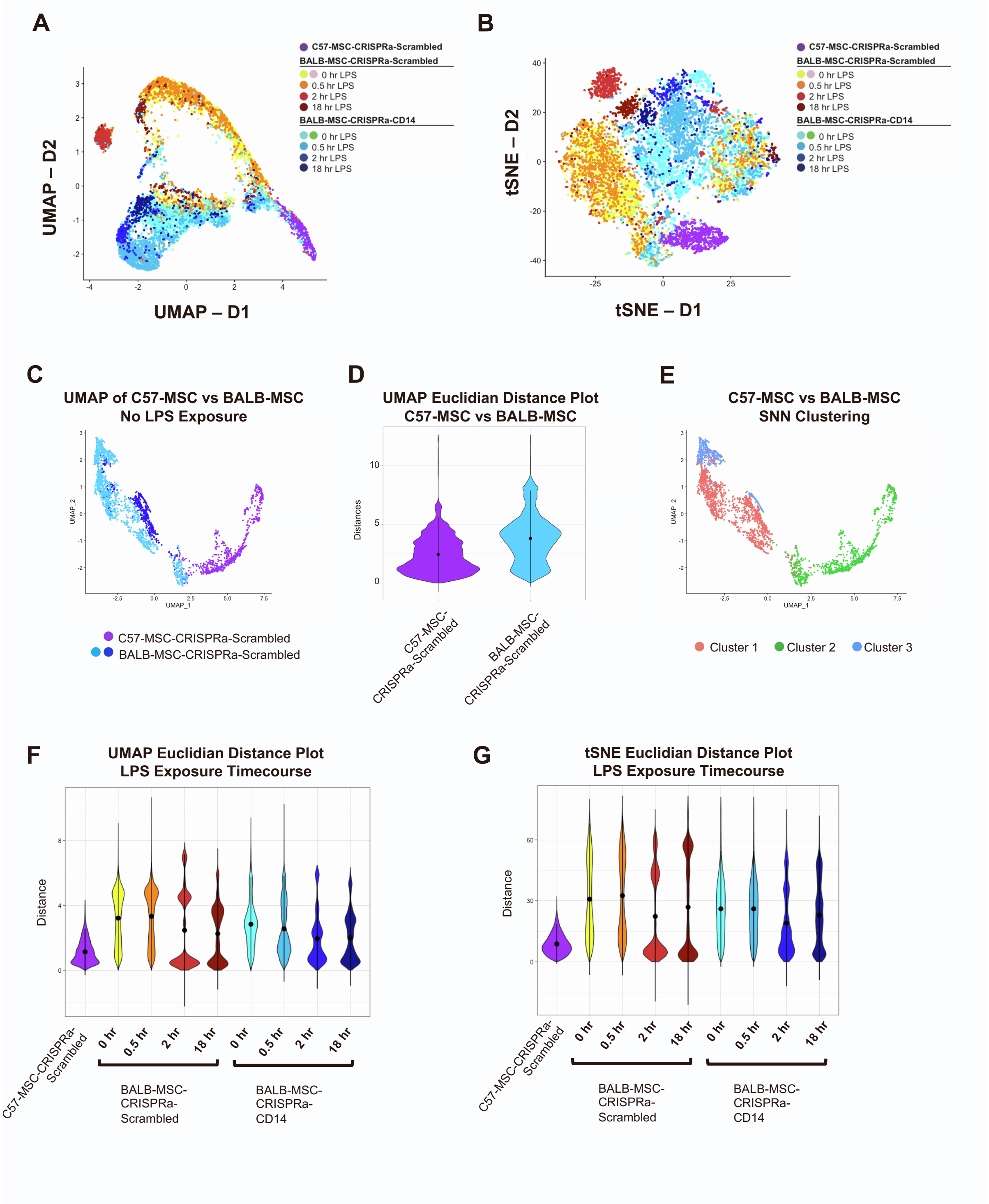

Figure S11. Single-cell RNA-seq visualizations and Euclidean distance plots, related to Figure 5. (A and B) Population structure visualization of single-cell RNA-seq profiles from MSCs during LPS-exposure time course using UMAP and tSNE

dimensionality reduction methods. **(C)** UMAP plot depicting single cell transcriptional profiles of untreated C57-MSC-CRISPRa-Scrambled and BALB-MSC-CRISPRa-Scrambled cells distribution of transcriptomic cell states without LPS treatment or CD14 addition. **(D)** Violin plot depicting pairwise n-dimensional Euclidian distances (with n being number of genes) between all cells in the UMAP projection from panel C within each cell type. A Kruskal-Wallis non-parametric rank sum test shows a significance of $p < 2.2e-16$ with a chi-squared statistic of 141984, suggesting that C57-MSCs have more closely related transcriptional profiles within the population compared to BALB-MSCs. **(E)** Seurat's shared nearest neighbors (SNN) algorithm was used to identify clusters of cells. Using a resolution value set at 0.1 we observed all C57-MSCs clustered together and BALB-MSCs were spread across three clusters (with some cells overlapping with the C57-MSC cluster). **(F and G)** Violin plot depicting pairwise n-dimensional Euclidian distances during LPS-exposure between members of the same experimental sample for both **(F)** UMAP **(G)** tSNE approaches.

Primer Name	Sequence
CD14-7-F	CACCGGTACGCACCAGACAAGTCCG
CD14-7-R	AAACCGGACTTGTCTGGTGCGTACC
CD14-49-F	CACCGGAATAATGATCTAAGGCACT
CD14-49-R	AAACAGTGCCTTAGATCATTATTCC
CD14-83-F	CACCGGAAAATGGAGGTGAATCAAT
CD14-83-R	AAACATTGATTCACCTCCATTTTCC
CD14-105-F	CACCGTTGCTAGCAACTAAGACTAG
CD14-105-R	AAACCTAGTCTTAGTTGCTAGCAAC
CD14-131-F	CACCGAAGAGCTGGATTTGAACGGT
CD14-131-R	AAACACCGTTCAAATCCAGCTCTTC
CD14-160-F	CACCGTGAATGTAATTGGACATTTG
CD14-160-R	AAACCAAATGTCCAATTACATTCAC
TLR4-8-F	CACCGCAGATCGTCATGTTCTCTCA
TLR4-8-R	AAACTGAGAGAACATGACGATCTGC
TLR4-32-F	CACCGTGGTGGCAGCGCAGAGTCCC
TLR4-32-R	AAACGGGACTCTGCGCTGCCACCAC
TLR4-53-F	CACCGAGGGAAGAGGCAGGTGTCCC
TLR4-53-R	AAACGGGACACCTGCCTCTTCCCTC
TLR4-76-F	CACCGCTTGCAGAGGGGCACCCACT
TLR4-76-R	AAACAGTGGGTGCCCCTCTGCAAGC
TLR4-126-F	CACCGAACCTTAGCATTCTCACTTT
TLR4-126-R	AAACAAAGTGAGAATGCTAAGGTTC
TLR4-159-F	CACCGGAATCGATCTGCCCCGTCGC
TLR4-159-R	AAACGCGACGGGGCAGATCGATTCC
NonTargetingControl_0001_F	CACCGGCGAGGTATTCGGCTCCGCG
NonTargetingControl_0001_R	AAACCGCGGAGCCGAATACCTCGCC
NonTargetingControl_0002_F	CACCGGCTTTCACGGAGGTTCGACG
NonTargetingControl_0002_R	AAACCGTCGAACCTCCGTGAAAGCC

NonTargetingControl_0003_F	CACCGATGTTGCAGTTCGGCTCGAT

Table S1. Primers used in this study, related to Figure 5.
